# QTL mapping and molecular characterization of the classical *D* locus controlling seed and flower color in *Linum usitatissimum* (flax)

**DOI:** 10.1038/s41598-017-11565-7

**Published:** 2017-11-16

**Authors:** Gurudatt Pavagada Sudarshan, Manoj Kulkarni, Leonid Akhov, Paula Ashe, Hamid Shaterian, Sylvie Cloutier, Gordon Rowland, Yangdou Wei, Gopalan Selvaraj

**Affiliations:** 1 National Research Council of Canada, 110 Gymnasium Place, Saskatoon, SK S7N 0W9 Canada; 2 Agriculture and Agri-Food Canada, 960 Carling Avenue, Ottawa, ON K1A 0C6 Canada; 3 Crop Development Centre, Department of Plant Science, University of Saskatchewan, Agriculture Building, 51 Campus Drive, Saskatoon, SK S7N 5A8 Canada; 4 Department of Biology, University of Saskatchewan, 112 Science Place, Saskatoon, SK S7N 5E2 Canada; 5 Present Address: Bayer CropScience, Crop Analytics Morrisville, TECHIII 407 Davis Drive, Morrisville, NC 27560 USA

## Abstract

The flowers of flax (linseed) are blue-hued, ephemeral and self-pollinating, and the seeds are typically brown. A century-old interest in natural yellow seed variants and a historical model point to recessive alleles in *B1*, *D* and *G* loci being responsible, but the functional aspects had remained unknown. Here, we characterized the “*D*” locus by quantitative trait loci (QTL) mapping and identified a *FLAVONOID 3*′5′ *HYDROXYLASE (F3*′5′*H)* gene therein. It does not belong to the F3′5′H clade, but resembles biochemically characterized F3′Hs (flavonoid 3′ hydroxylase) but without F3′H activity. The genome lacks other *F3*′*H* or *F3*′*H*-like genes. The apparent neo-functionalization from F3′H is associated with a Thr_498_ → Ser_498_ substitution in a substrate recognition site (SRS). The yellow seed and white flower phenotypes of the classical *d* mutation was found to be due to one nucleotide deletion that would truncate the deduced product and remove three of the six potential SRS, negatively impacting delphinidin synthesis. Delphinidin is sporadic in angiosperms, and flax has no known pollination syndrome(s) with functional pollinator group(s) that are attracted to blue flowers, raising questions on the acquisition of F3′5′H. The appearance of *d* allele is suggestive of the beginning of the loss of F3′5′H in this species.

## Introduction

Flax is the seed/fiber crop among the eight founder crops in agriculture^1^. Archeological evidence indicates use of flax fibers nearly 30,000 years ago^2^. Flax offers the richest seed source of 18:3, Δ^3–5^ α-linolenic acid (ALA; ω–3 fatty acid). Oxidative polymerization of ALA renders flax oil its characteristic drying property that is capitalized upon in inks, varnishes and paints. ALA also has health-promoting properties that have made flax products popular nutritional supplements^6^. However, ALA-rich oil has poor shelf life; it becomes rancid and is unusable as staple oil. In Australia and Canada, breeding efforts were undertaken to enable kitchen use of flax oil by genetically reducing the ALA content by incorporating mutations in fatty acid desaturation^7,8^. In Canada, the largest exporter of flax seed in the world, it became mandatory to segregate low-ALA flax for trade purposes. Yellow seed color was adopted to distinguish low-ALA types from the mainstream high-ALA flax commodity which is brown-seeded. The subsequent lack of markets for low-ALA varieties triggered the de-registration of all low-ALA flax varieties in Canada. Consequently, the exclusive use of yellow seed color for low-ALA type has become unnecessary and yellow seed variety with high-ALA can now be produced. Yellow seeds are generally larger, and contain more protein and oil^9,10^. This is attributed to altered seed coat properties. The world’s germplasm collections comprise 4.3% yellow seed types^9^. Thus, breeding yellow seed varieties for mainstream industrial purposes has become an attractive proposition.

There has been a longstanding interest in the genetics of flax seed types^11^. Three loci (*B1*, *D* and *G*) when in recessive state were postulated to result in yellow seed color^11^ and subsequently a dominant locus/allele (*Y or Y1*) was also found^3,12^. All four loci were proven to be independently inherited^13^. Arabidopsis *tt* (*transparent testa*) mutants defective in PA biosynthesis have been studied extensively. Yellow seed color is a result of blocked biosynthesis of proanthocyanidins (PA; condensed tannins) that impart the brown color to the seed coat^4,5,14,15^. While yellow flax seeds are desirable for their oil content, the lower PA content also offers some advantages. The seed coat is not removed during oil extraction and thus the defatted flax seed meal, which is a protein-rich supplement in animal feed, contains PA that negatively affects protein digestion^16^. Low-PA meal is preferred in animal feed supplements^17,18^. PA biosynthesis in flax has not been studied at the molecular level.

Despite the longstanding interest in flax seed and flower color, there have been no reports on the genes underpinning the phenotypes. This work reveals the genetic, biochemical, molecular and evolutionary aspects of the formation of the classical blue flowers and brown seeds in flax; it shows how flax differs from model systems such as Arabidopsis, and raises some questions about the gain of the capacity to make delphinidin at the expense of cyanidin biosynthesis.

## Results

### The *d* mutant accumulates much less PA in the endothelium resulting in yellow seed color

**Figure 1:**
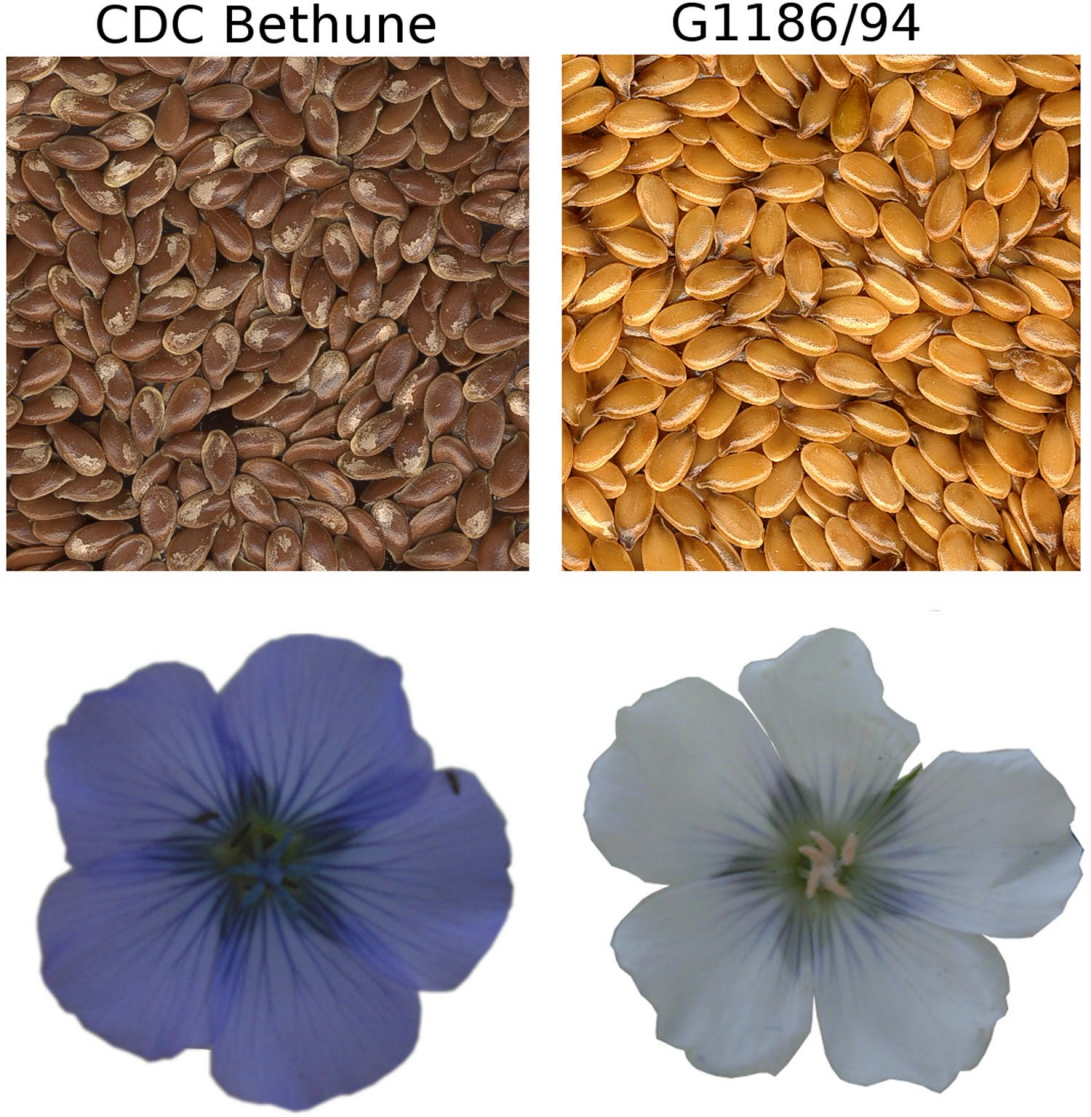
Seed and flower color phenotypes of CDC Bethune (a representative of the wild type; brown seed and blue-hued petals) and G1186/94 (*d* mutant with yellow seed and white petals).

**Figure 2:**
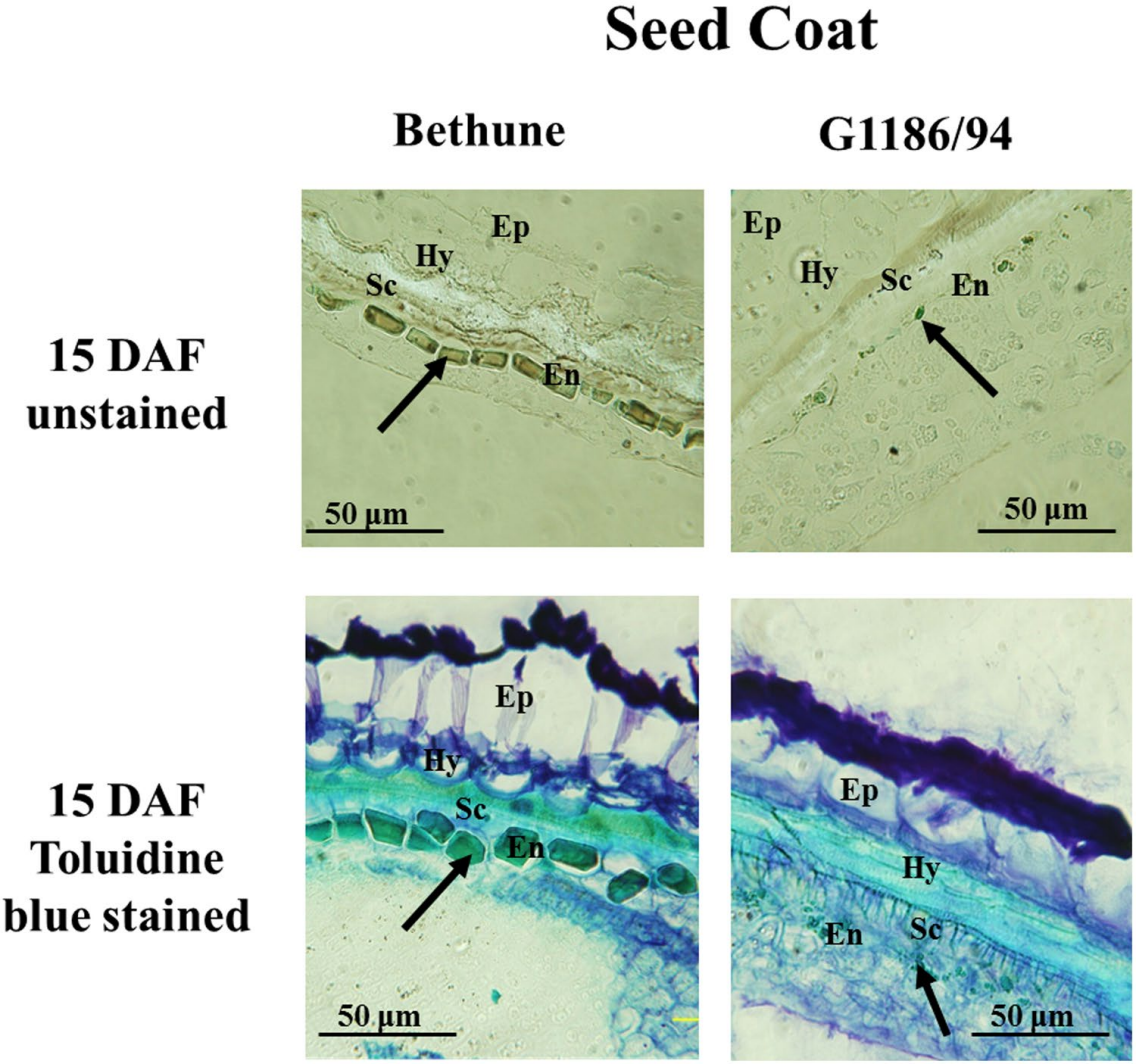
Deficiency in pigment accumulation in the seed coat of G1186/94 (yellow seed) in contrast to CDC Bethune (brown seeds) as shown in cross sections of seeds at 15 days after flowering (DAF). The two upper panels are unstained sections, and the two bottom panels are sections stained with toluidine blue. The dark coloration in both the unstained and stained CDC Bethune endothelium is indicative of the presence of proanthocyanidins (PA) as indicated by the arrows. The corresponding cell layer in G1186/94, which is deficient in PA accumulation, is also indicated by arrows. (Ep- epidermis, Hy- hypodermis, Sc-sclerenchyma layer, En- endothelium).

CDC Bethune (*B1B1, DD, GG* genotype)^19^ has brown seeds and blue petals, as most of the global flax accessions do, whereas G1186/94 (*B1B1, dd, GG*)^13^ has yellow seeds and white petals (Fig. 1). As elucidation of the molecular basis of the *d* allele and its impact on seed color was a major objective of this study, our initial work concerned seed analysis for PA accumulation. Comparative phytochemistry of developing seeds of CDC Bethune indicated that seeds began accumulating PA at 6 days after flowering (DAF) (L. Akhov, unpublished). As shown in Fig. 2, the endothelium in the toluidine blue-stained sections of seeds of 15 DAF showed differences in the two genotypes. Endothelial cells of G1186/94 were smaller in size as compared to CDC Bethune, indicating perturbed development of this seed coat layer. The unstained sections, the endothelium in CDC Bethune but not in G1186/94 was brown, which we attribute to PA accumulation.

**Figure 3:**
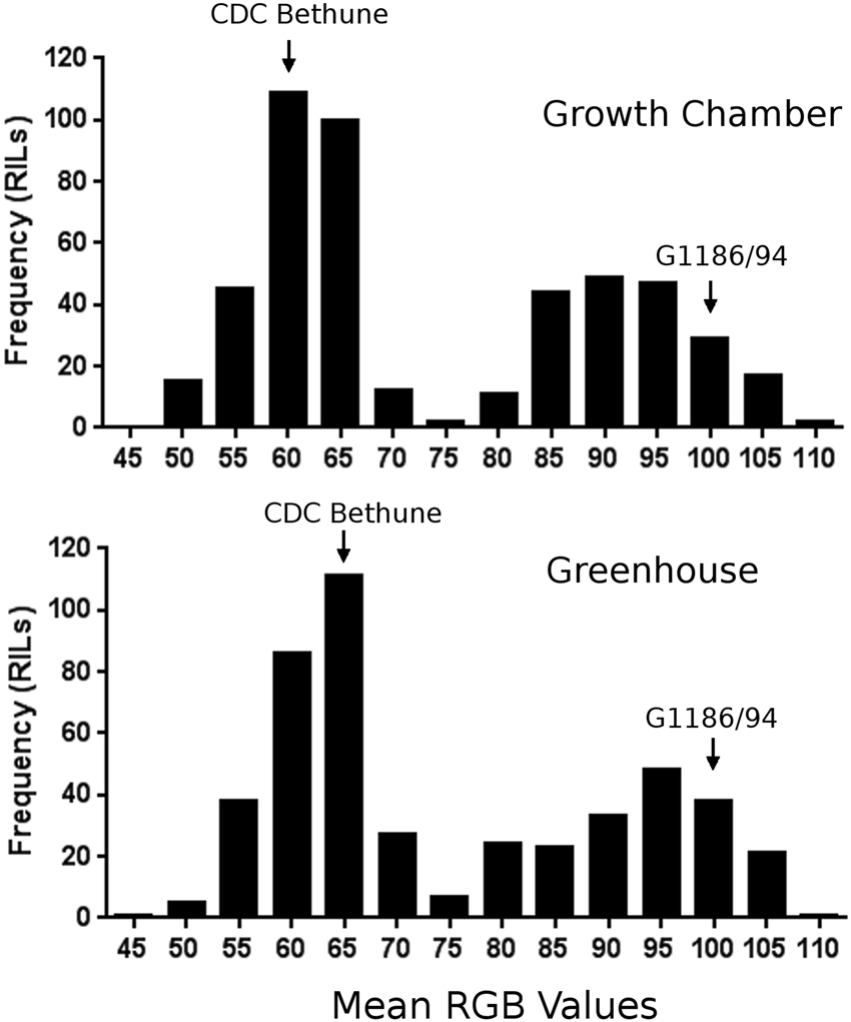
Phenotypic distribution of seed color in G1186/94 X CDC Bethune recombinant inbred line (RIL) population grown in two different environments (a controlled growth cabinet and a less controlled greenhouse). Brown seeds (e.g. CDC Bethune) had a lower mean RGB value of <70 whereas yellow seeds (e.g. G1186/94) had a value of >80. The parental lines indicated by arrows had the phenotypes shown in Fig. 1.Transgressive segregants for both darker brown and brighter yellow were evident in both environments.

Seed color was quantitatively evaluated in the red (R), green (G) and blue (B) spectrum through scanning. The brown seeds of CDC Bethune had an RGB value of 56.7 ± 0.4 whereas the yellow seeds of G1186/94 had a value of 99.3 ± 3.0 when the lines had been grown under controlled conditions in a growth cabinet. The values in a somewhat more variable greenhouse environment were also distinct for the brown and yellow seeds: 63.5 ± 4.5 for CDC Bethune and 99.7 ± 3.1 for G1186/94. The correlation between the two environments was 91.7%. The F_8:9_ and F_9:10_ recombinant inbred lines (RIL) derived from these parents showed a bimodal distribution of the phenotypes **(**Fig. 3), indicative of a single major locus, in agreement with Mittapalli and Rowland^13^ who observed a 3:1 brown to yellow ratio in F_2_-derived seeds of three G1186/94 (female) by brown seeded lines (male) crosses.

### Mapping the *d* allele indicates the presence a putative *FLAVONOID 3*′ *HYDROXYLASE* (*F3*′*H*) gene of CYP75B type

**Figure 4:**
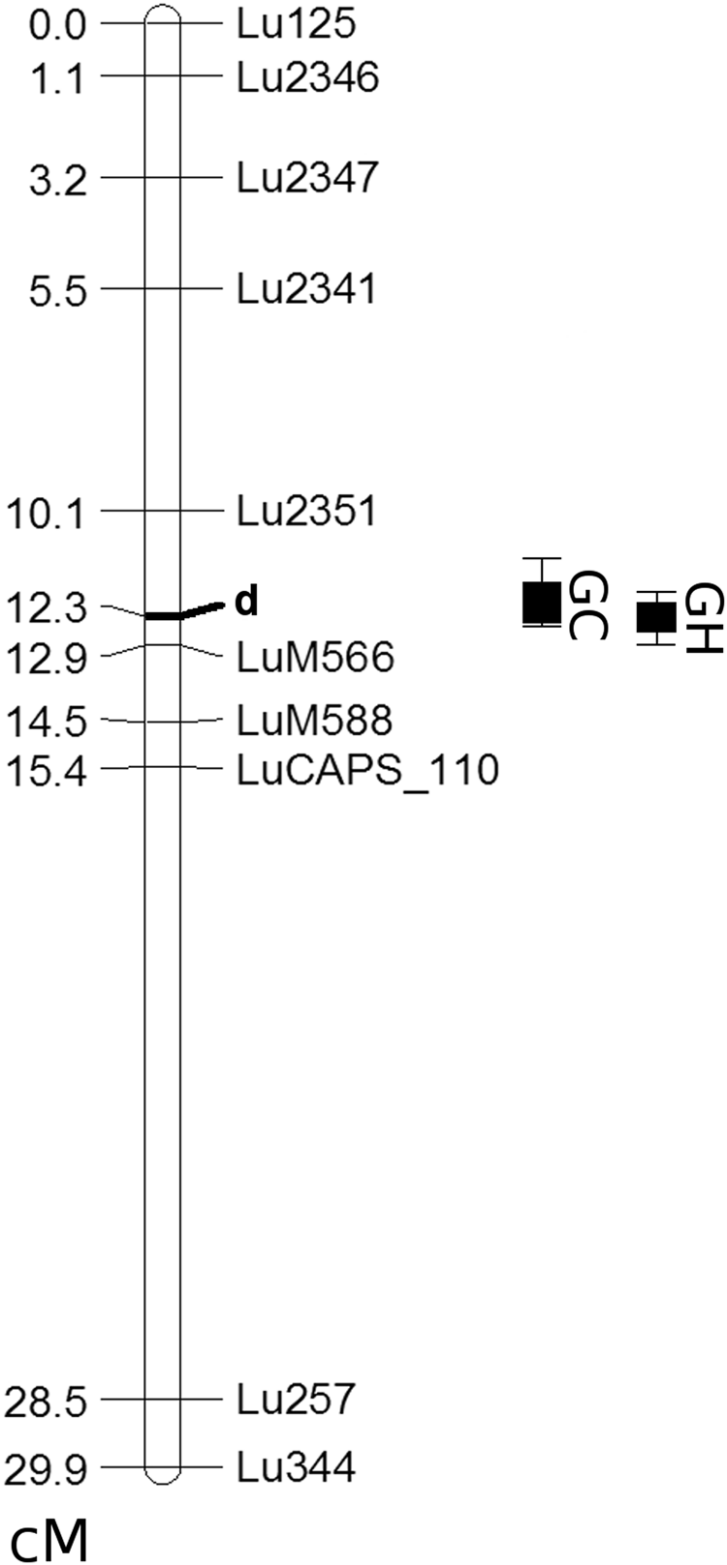
Fine mapping of the ***D*** locus in LG2. Recessive mutation *d* imparts both yellow seed and white petal color. QTL location between Lu2351 and LuM566 in recombinant inbred line population of G1186/94 x CDC Bethune grown in two environments is shown: GC, controlled growth cabinet (LOD 47.1 and R^2^ 0.89); GH, greenhouse (LOD 39.1 and R^2^ 0.84). The genetic location of some markers are not shown in the figure to avoid crowding the depiction, but it is indicated in parentheses, preceding the physical location corresponding to the nucleotide sequence of Scaffold 208^24^: LuM566 (12.9), 53.5 kb; LuM568 (12.9), 58.5 kb; LuM569 (12.9), 58.6 kb;. LuM588 (14.5), 141.2 kb; LuM592 (14.5), 152.5 kb; LuM193 (14.5), 155.1 kb; LuM595 (14.5), 155.6 kb; LuM597 (14.5), 159.9 kb; Lu209 (14.5), 184.1 kb; LuM71 (15.4), 423.5 kb; LuCAPS_110, 447.7 kb. The primer information for the LuM SSR markers is provided in Supp. Table S1. LuCAPS_110 is a CAPS marker that is described in Methods. (cM = centimorgans).

A genetic map of the CDC Bethune x G1186/94 RIL population based on SSR markers spanning all 15 linkage groups (LGs) of flax was constructed. Initially, 47 brown and 47 yellow seed recombinant inbred lines were mapped with SSR markers **(**Supp. Table S1**)**. This resulted in a framework genetic map comprising 83 markers spanning a total genetic distance of 467.8 cM and 19 LG (Supp. Fig. S1). LG1 named here corresponds to LG2 of the published integrated genetic and physical map of flax^20,21^ and it harbours the *D* locus based on the QTL analysis for seed color. Additional markers were developed in the interval to fine-map the QTL and markers explaining the 84 to 89% of the phenotypic variance were identified (Fig. 4).

The SSR markers Lu209 and Lu125 that were initially found to be linked to the *d* allele were derived from ESTs^22^. The SSR in Lu209 corresponds to two nested ESTs of otherwise identical sequences: Accession No. JG040203.1; JG047082.1^22,23^. A BLAST query (e-10) of the flax reference genome sequence^24^ located the ESTs on Scaffold 208 which is 830.6 Kbp. All subsequent markers for fine-mapping were derived from this scaffold sequence by in silico scanning for potential SSRs. Of the 52 potential SSR markers identified 8 were experimentally found to be polymorphic between CDC Bethune and G1186/94 **(**Supp. Table S1**)**. In addition, mapping Illumina short reads from the re-sequencing of G1186/94 identified SNPs from which one CAPS marker was developed. Mapping these 9 markers on the RIL population helped identify the terminal 53.5-kb region of Scaffold 208 as the region of interest in the *D* locus to find the *d* allele (Fig. 4).

Scaffold 208 sequence was subjected to FGENESH prediction with *Hevea* for gene model^25^, and predicted amino acid sequences were used for BLAST against the TAIR database. A total of 236 genes were predicted **(**Supp. Table S2**)**. The 53.5 Kb region of Scaffold 208 where the *D* locus had been mapped has 14 candidate genes (Supp. Table S3**)**. One of these is annotated as a putative F3′H gene between 7127–9095 bp of the scaffold (*Lus10021620* in the flax genome; https://phytozome.jgi.doe.gov/pz/portal.html#!info?alias=Org_Lusitatissimum). The predicted gene has three exonic regions that code for 521 amino acids that have 61.7% identity with the deduced F3′H polypeptide of the *Arabidopsis TRANSPARENT TESTA 7* (*TT7*) gene (AT5G07990.1) over the entire length. The latter is a CYP75B subfamily type in the CYP71 clan of cytochrome P450 monooxygenases^26^.

### Phytochemistry indicates absence of F3′H activity in flax and instead reveals 3′5′ hydroxylation that is defective in G1186/94 in seeds and petals

**Figure 5:**
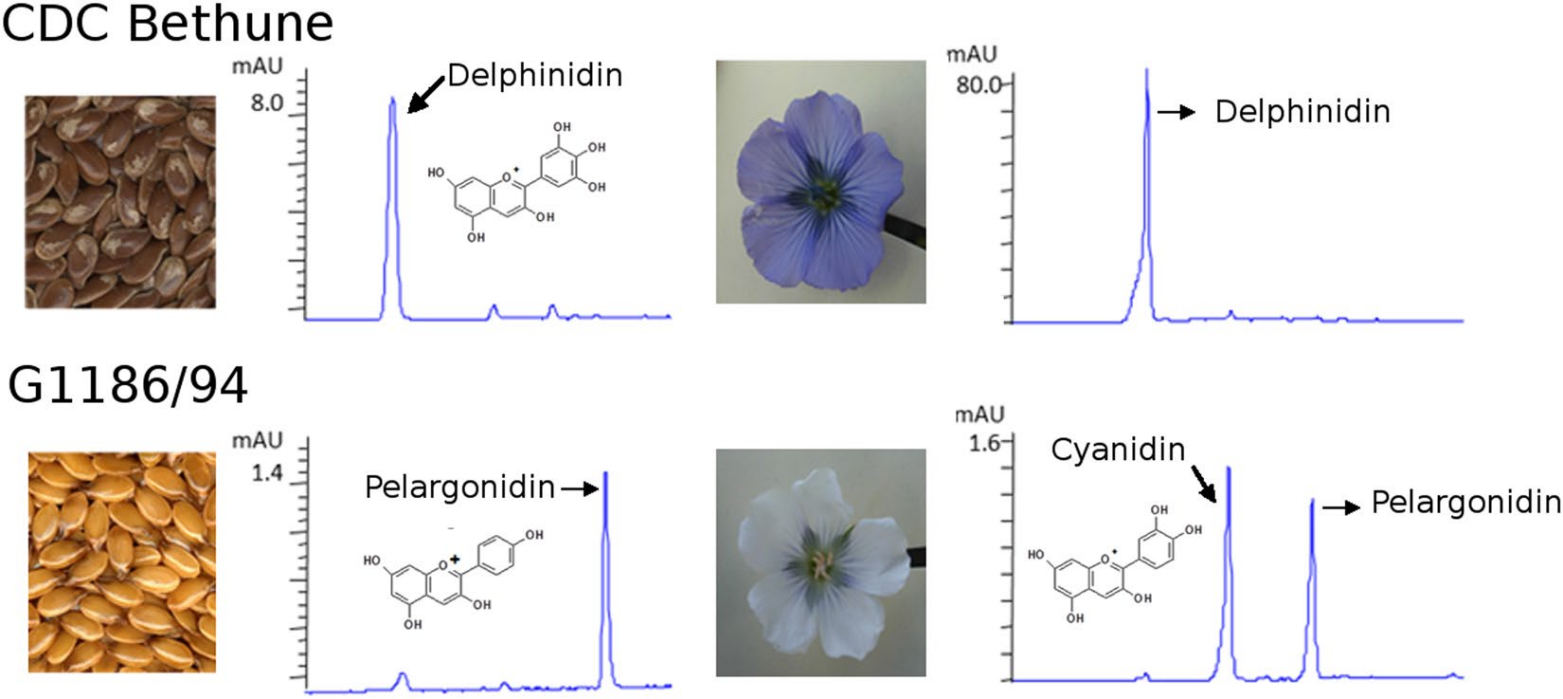
Qualitative and quantitative differences in the flavonoids from seeds and petals of CD Bethune and G1186/94 as shown by HPLC profiles of acidified butanol extracts. Note the compressed scale for CDC Bethune and expanded scale for G1186/94. CDC Bethune flowers also contained a similar amount of cyanidin as in G1186/94 flowers.

Polymerized PA is generally recalcitrant to compositional analysis. Acidified butanol extraction is a commonly used method to analyze the polyflavan structure after partial de-polymerization^27^. As a result of this extraction, flavans are transformed into corresponding anthocyanidins. The subsequent anthocyanidins serve to deduce the B-ring hydroxylation pattern(s) in PA. Our analyses showed that the seed coats of CDC Bethune contained delphinidin (3′4′5′ hydroxylated) and only trace levels of cyanidin (4′5′ hydroxylated). G1186/94 seed coat extracts contained very low levels of pelargonidin (4′ hydroxylated) but not delphinidin, indicating defective 3′5′ hydroxylation **(**Fig. 5**)**. The trace level of cyanidin in G1186/94 was comparable to that in CDC Bethune. Thus, gallocatechin (3′4′5′ trihydroxy flavan-3-ol) in CDC Bethune and afzelechin (4′hydroxy flavan-3-ol) in G1186/94 are the main components of seed coat PA.

CDC Bethune petals (blue) had delphinidin whereas G1186/94 flowers (white) did not. The latter contained very low amounts of pelargonidin that was not detected in seeds and petals of CDC Bethune. The lower levels of pelargonidin in G1186/94, relative to the delphinidin content in CDC Bethune derived from dihydromyricetin (3′,4′,5′ hydroxylated), suggest that the next step in the pathway after B-ring hydroxylation mediated by dihydroflavonol-4 reductase (DFR) is less efficient in accepting dihydrokaempferol (4-hydroxylated). The lack of delphinidin anthocyanins in G1186/94 explains the white petal phenotype. The smaller amounts of afzelechin based PA in G1186/94 might not be sufficient to impart a brown seed phenotype.

The F_8:9_ RIL population comprising 479 lines showed that all brown-seeded lines (280) had blue petals and all yellow-seeded lines (199) had white petals. We then phytochemically analyzed a random subset of 67 blue-petal lines and 33 white-petal RILs. The chemotypic characteristics of all blue-petal lines were like the above-noted CDC Bethune type and that of all white-petal lines were like G1186/94. These results collectively showed that while the best candidate gene in the *D* locus was a putative *F3*′*H* gene, the biochemical defect in *d* mutant was in 3′5′hydroxylation and consequently the gene was presumed to encode a 3′5′ hydroxylase. F3′5′H directs the flavonoid pathway to the delphinidin branch and mutations affecting expression of the *F3*′5′*H* gene impact delphinidin synthesis^28,29^.

### Downregulation of the putative *FLAVONOID* 3′5′ *HYDROXYLASE* in G1186/94 seeds and petals supports the chemotypic deviation from CDC Bethune

**Figure 6:**
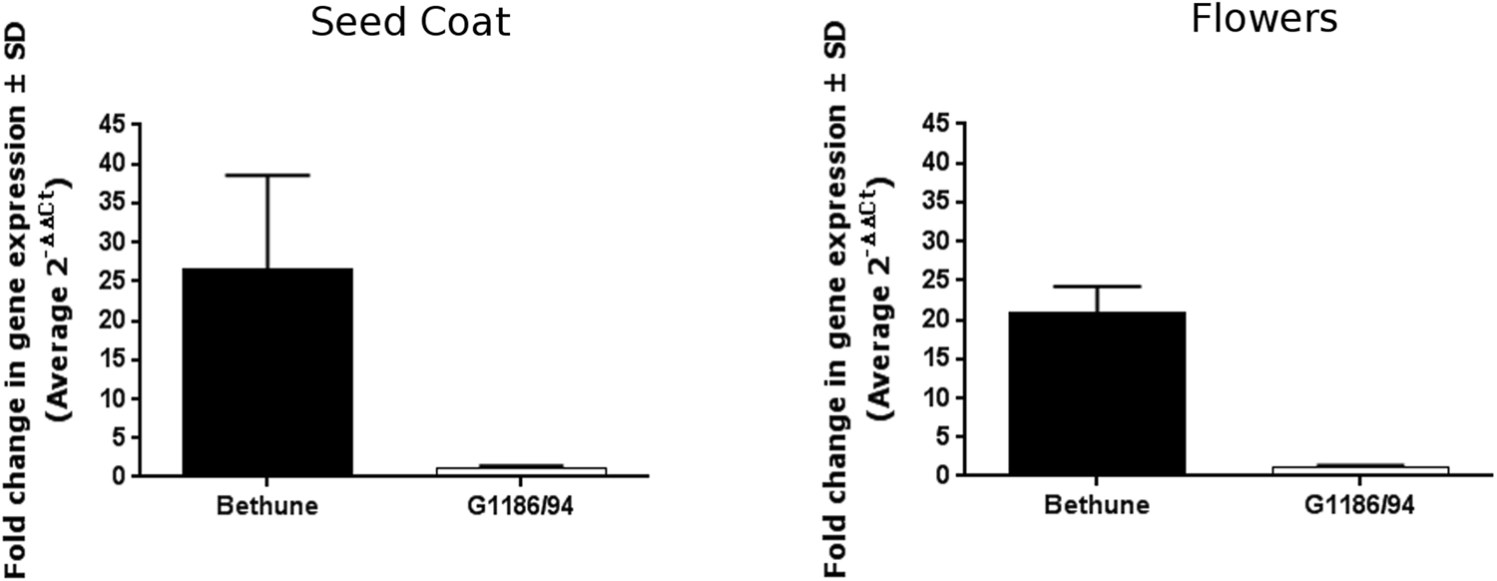
Lower expression of *F3*′5′*H* gene in G1186/94 as identified from qRT-PCR analysis of transcripts in seed coat (15 days after flowering) and petals (flower buds at anthesis before opening). EF1α was used as the common internal control reference gene^24^. Higher level of expression in CDC Bethune relative to G1186/94 is shown.

The predicted coding sequence from Scaffold 208 as a query against all ESTs of *Linum* in Genbank identified only six short-sequence ESTs among the 286,900 entries in the database. All these six ESTs were from CDC Bethune^23^: One from seed coat tissue corresponding to torpedo stage embryo; two from flower tissues and three from endosperm tissue. One of these 6 ESTs (286 bp) represented the 5′ end; the other 5 were at the 3′ end, four of them were shorter and nested under the longest sequence of 404 bp with 100% identity **(**Supp. Fig. S2**)**. We performed quantitative gene expression analysis (qRT-PCR) of seed coat tissue and flower petals in CDC Bethune and G1186/94. CDC Bethune had ~20-fold higher expression in petals and ~25-fold higher expression in seed coat tissue relative to the expression levels in G1186/94 (Fig. 6). The results established that the diminished expression of the putative *FLAVONOID 3*′5′*HYDROXYLASE* gene in *d* allele in G1186/94 impacted both seed and petal color.

### Loss of flavonoid 3′5′hydroxylase function in G1186/94 is due to a premature translational stop mutation in a gene resembling *F3*′*H*

**Figure 7:**
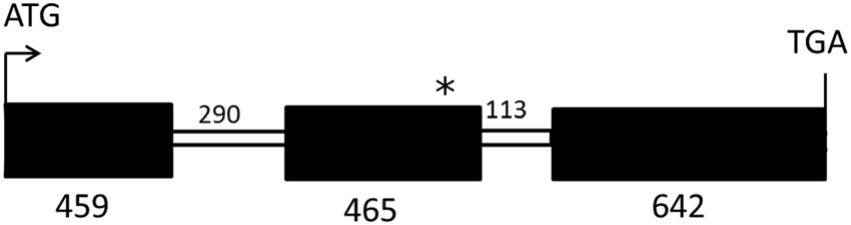
Depiction of *F3*′*5*′*H* gene in CDC Bethune and G1186/94. Exons are shown as filled boxes and introns as open boxes. The number of nucleotides in each of these is as indicated. The asterisk denotes a premature stop codon created due to a single nucleotide deletion in G1186/94. Further details are in Supplementary Figures 3 and 4. The sequence between ATG and TGA shown in the figure was amplified with suitable primers highlighted in Supplementary Figure 3. An alignment of CDC Bethune and G1186/94 amplicon sequences is shown in Supplementary Figure 4.

Re-sequencing the six ESTs fully showed that these clones contained only the short sequences reported in Genbank. Full-length genomic sequences from CDC Bethune and G1186/94 were retrieved by PCR with forward and reverse primers designed from the sequences of the ESTs JG242995.1 and JG079443.1, respectively. PCR errors were ruled out by five replicates of PCR. The 1969-bp sequence encompassing exons and introns were identical in CDC Bethune and G1186/94 (Fig. 7) except for a single nucleotide deletion in the second exon of G1186/94 (Supp Fig. S3). This deletion would create an in-frame stop codon prematurely terminating the polypeptide after 278 amino acids in G1186/94, whereas the open reading frame in CDC Bethune would encode a polypeptide of 521 amino acids (Supp. Fig. S4). Notably, a subsequent re-sequencing of Avantgard, the donor of the *d* allele in G1186/94, also confirmed the occurrence this single nucleotide deletion (Supp. Fig. S5).

### The *F3*′5′*H* gene is a later acquisition of dual hydroxylation function from *F3*′*H*

**Figure 8:**
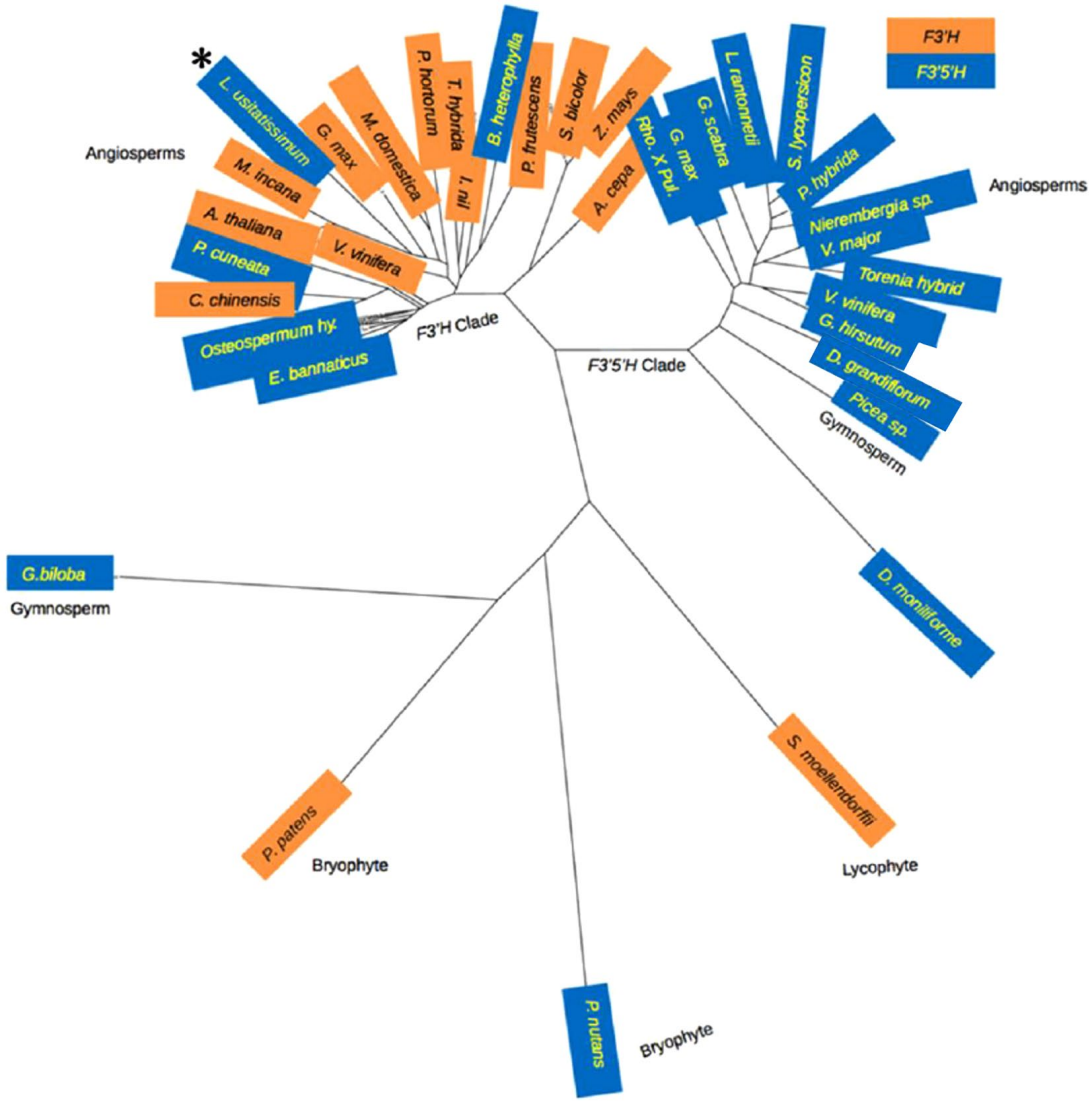
Location of the flax F3′5 H (marked with an asterisk) in the F3′H clade. The unrooted phylogenetic tree was constructed as described in Methods. The accessions are from BRENDA (http://www.brenda-enzymes.org), Seitz *et al*.^31,32^ and NCBI. The species names and the entries are as in Fig. 9. The enzymatic function is known for all entries except those from *Ginkgo biloba, Picea glauca, Physcomitrella patens, Pohlia nutans, Selaginella moellendorffii*.

**Figure 9:**
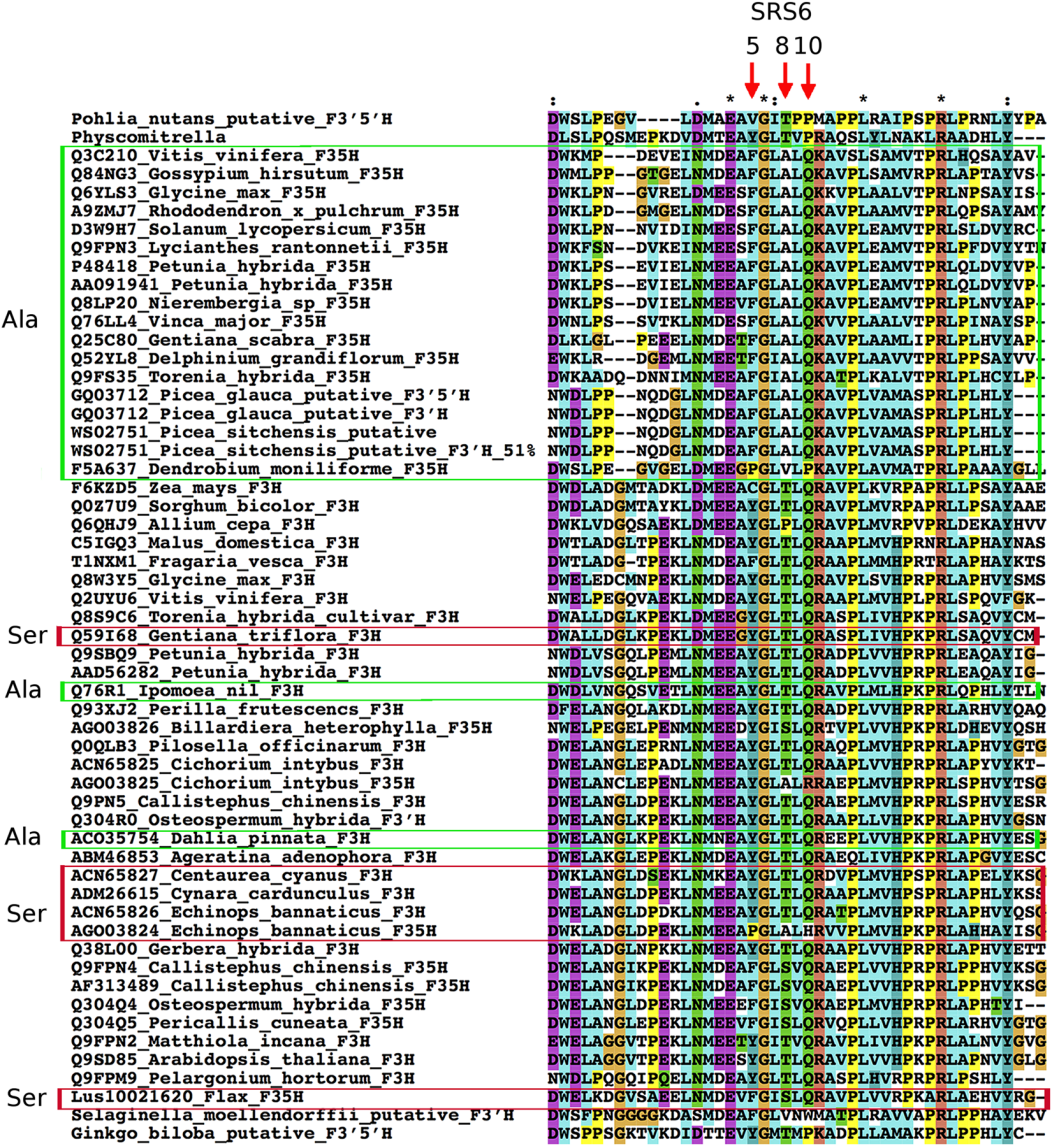
Alignment of putative F3′H and F3′5′H amino acid sequences with marked region for substrate recognition site (SRS6). The accessions are from BRENDA (http://www.brenda-enzymes.org), Seitz *et al*.^31,32^ and NCBI. The positions 5, 8 and 10 within SRS6 are indicated by arrows. Notable for substrate specificity is position 8^31,32^. The accessions where a Ser (S) is present at this site instead of Thr (T) are boxed in red; Ala (A) substituted sequences are boxed in green. These boxed proteins are F3′5′H whereas others are F3′H. “Lus10021620_Flax_F35H” boxed in red is the flax *D* locus gene product that has a Ser substitution at position 8.

The biochemical defect seen *in vivo* being a loss of F3′5′H rather than F3′H activity was resolved from sequence analysis. The flax F3′5′H (521 amino acids) is 64% identical over 97% of its sequence to the biochemically characterized grape F3′H (BAE470005.1; ALP48438.1 and NP_001267916.1). In a phylogenetic analysis using the Neighbour-Joining method, it did not cluster with classical F3′5′H enzymes but instead grouped with the F3′H clade. To avoid any uncertainty of enzymatic function from tentative annotations of sequences in databases, we repeated the analyses with only biochemically characterized F3′H and F3′5′H and these indicated clearly that the flax sequence is more related to F3′H than to F3′5′H (Fig. 8). Taking the grape F3′H amino acid sequence as a reference, the flax F3′5′H sequence has at its 498^th^ amino acid position (487 in grape) a serine (Ser) residue instead of a threonine (Thr). Earlier research^30–32^ reported that such a substitution in *Gerbera hybrida* converts F3′H to F3′5′H and, the reverse of it in *Osteospermum hybrida* F3′5′H makes it an F3′H enzyme. Not only does replacement of Thr with Ser make this phenomenal switch but also Ala at this position is associated with 3′5′ hydroxylation function (Fig. 9)^33^. In addition to the flax F3′5′H, only four other F3′5′H that bear more structural resemblance to F3′H have been identified to date^32^. Three are in Asteraceae and one from Pittosporaceae (Fig. 9). These independently derived, neo-functionalized enzymes represent those appearing late in evolution.

## Discussion

CDC Bethune provides a typical example for the preponderance of blue flower and brown seed types in global flax accessions^13^. G1186/94 represents an ancient naturally occurring variant whose seed is yellow and flower petals are white^13^. In a classical study of yellow seed variants, an un-mutated form of the genetic factor *D* has been shown to make flowers lilac and also to intensify the color whereas the recessive *d* version renders flowers pale/pink^11^. The binary chemotypic distinction of the brown-seed, blue-flower lines from the yellow-seed, white-flower lines among the stable single-seed descendants of genetic crosses between CDC Bethune and G1186/94 establishes a common genetic event underlying the phenotypic contrast. We have mapped the major QTL associated with seed and flower color and have delineated the molecular characteristic of the *d* allele. We have shown that the *d* allele carries a single nucleotide deletion in the *F3*′5′*H* gene causing a frameshift and premature termination, resulting in yellow seeds and white flowers. Prior modeling studies had identified six potential substrate recognition sites (SRS) in P450 monooxygenases^34,35^. The premature termination codon in the *d* allele would remove SRS 4, 5 and 6 in the gene product. The 20- to 25-fold lower level of transcripts in G1186/94 might be due to nonsense-mediated decay (NMD) of transcripts, as seen in an *Ipomea purpurea* mutant where the deduced polypeptide of *F3*′*H* is truncated by 241 amino acids due to a premature stop codon^36^.

The *FLAVONOID 3*′5′ *HYDROXYLASE* gene itself is a recent acquisition in the evolution of flowering plants, where the capacity to perform dual hydroxylation (3′5′-hydroxylation) on the flavonoid B-ring has arisen by a seemingly conservative substitution of Thr to Ser while simultaneously losing the ability for 3′-hydroxylation. The phylogenetic relationships clearly establish a closer association to the F3′H clade than to F3′5′H clade. Only trace levels of cyanidin were found in G1186/94, and these were not higher in CDC Bethune despite the presence of an intact *F3*′5′*H* gene in the latter. We found in the flax genome^24^ neither an additional *F3*′*H-*like nor an *F3*′5′*H*-like gene in BLAST analyses (M. Kulkarni, unpublished). This indicates the incapability of the flax flavonoid 3′5′ hydroxylase for 3′ monohydroxylation alone to any significant extent. Seeds and petals as contrasting tissue types that generally synthesize flavonoids show very little cyanidin, and these small amounts present in the seeds and flowers is not due to a *bona fide F3*′*H* gene. We surmise that it is due to “metabolic noise” due to substrate promiscuity of some P450 enzymes^37^. For instance, metabolomic analyses have documented the presence of some substances in Arabidopsis for which no established biosynthetic routes exist or can be predicted from the genome sequence^38^.

Since lower plants contain 3′ and 3′5′ hydroxylated flavonoids, it is believed that the two hydroxylases diverged early in the evolution^39^. However, *F3*′*H* appears to be more extant than *F3*′5′*H* as evidenced by the scant appearance of the latter in plant taxa. The few examples of active *F3*′5′*H* hydroxylase genes in plant species include *Vitis*, *Petunia, Vinca, Gentiana, Solanum*, *Cichorium intybus*, *Echinops bannaticus*, *Pericallis cruenta*, Osteospermum, and Callistephus^32^. Many other ornamental species like rose, gerbera, lily, chrysanthemum and the fully sequenced model plant Arabidopsis lack *F3*′5′*H*^40^. Loss of *F3*′5′*H* due to spontaneous mutations is also evident in otherwise blue-flowered plants^41,42^. Assuming that *F3*′5′*H* has been lost many times^31,32,43,44^, sporadic re-appearance of it is intriguing.

The appearance of the flax *F3*′5′*H* gene within the *F3*′*H* clade suggests the presence of an *F3*′*H* gene in the ancestry of flax. How did flax *F3*′5′*H* arise? If it was by gene duplication, the genome should have another *F3*′*H*-like gene which is not the case; hence, suggesting neo-functionalization of a single copy gene at the expense of F3′H activity. Gene duplications relieve adaptive conflicts. Two models have been proposed: escape from adaptive conflict (EAC)^45^ and invention-amplification-diversification (IAD)^46^. The former postulates that duplication provides a mean to address adaptive conflict if an old function is compromised by a new function. The IAD model holds that some ancestral enzymes are as good as modern enzymes. Considering that flax F3′5′H is a modern enzyme, it is as good as the old enzyme of the classical F3′5′H clade in terms of delphinidin production. Note that EAC appears to apply to duplications of *DFR* genes whose function follows immediately after *F3*′*H* and *F3*′5′*H* in the flavonoid biosynthesis pathway^47^. The flax genome has two *DFR* genes that we have cloned (M. Kulkarni, unpublished). However, there is no gene duplication of *F3*′*H/F3*′5′*H* in flax despite the loss of the presumed ancestral F3′H activity suggesting that there was either no conflict from losing an antecedent F3′H function or that duplication occurred but was followed by subsequent gene loss that was not detrimental. The latter seems unlikely given the recent appearance of *F3*′*H*-like *F3*′5′*H* in flax.

F3′5′H is generally attributed to blue-hued flowers although blue color is not exclusively due to delphinidin anthocyanins^44^. The three F3′5′H enzymes of Asteraceae and the one from Pittosporaceae that arose from F3′H are considered to have afforded a selective advantage^31,32^. Why did flax (or its ancestor) gain F3′5′H at the expense of F3′H? Flower traits are shaped by pollination syndromes that encompass enticing floral characteristics and the predominant group(s) of pollinators^48^. White flowers for example tend to be self-pollinated whereas blue flowers attract bees and red flowers have more bird visits^43,49^. The literature indicates that delphinidin-based anthocyanins afforded on blue-hued flowers a pollination advantage by helping attract insects and that white flowers have become increasingly self-pollinating^50^. *L. usitatissimum* is a highly self-pollinating plant, and its flower is very short-lived, often lasting less than a day. Thus, there is no overt need in flax to have a blue flower with delphinidin. *L. bienne*, which is considered a progenitor of *L. usitatissimum*^51^, is also homostylous, suggesting an innate capacity for self-pollination even though bees and wasps visit its flowers. Keeping in mind the ancestry of flax is still unclear and that many *Linum* species are heterostylous that necessitates cross-pollination, the acquisition of F3′5′H in flax might pre-date the immediate ancestors. Its retention in flax suggests neutrality. Alternatively, the loss of it in the *d* allele might indicate the beginning of the loss of this function.

## Methods

### Plant materials

CDC Bethune (brown-seeded) is described in Rowland *et al*.^19^ G1186/94 (yellow-seeded) derived from Avantgard is from the flax breeding program of Prof. Dr. Wolfgang Friedt, University of Giessen, Germany^13^. Recombinant inbred lines (RILs) developed from a reciprocal cross between the parents were used for linkage mapping. The F_8:9_ seeds from RILs grown in a growth chamber (GC) were phenotyped for seed color. DNA extracted from single F_8:9_ grown in pots in a greenhouse (GH) was used for genotyping and the resultant F_9:10_ seeds were phenotyped for color.

### Microscopy

Seed samples were collected from flax capsules at several time points, defined as days after flowering (DAF), to represent important seed coat development stages. Tissue fixation, embedding and sectioning were performed as previously described^52^. Sections were stained with Toluidine-O-Blue 0.025% in aqueous solution. Seed development stages were followed as described^23^. Sections were observed and photographed using a Zeiss Axioplan (Germany) microscope. Pictures were taken with AxioVision4.7 software (Zeiss, Germany).

### Seed color measurement

For phenotyping, the RIL population of 479 lines of F_8:9_ seeds (GC) and 463 lines of F_9:10_ seeds (GH) were used. Flat bottom 96 Well Microtest^TM^ 96 well plates (BD Biosciences) were used to screen 8 seeds of each RIL as well as parental seeds. Seeds were scanned using an EPSON scanner (Expression 1680) at the 400 dpi pixel intensity using Silverfast (AiV6.22r4) software. Red = 129, Green = 125, Blue = 121, Luminosity = 126 and color value was set at 125 in Adobe Photoshop^R^ (Elements 9) software to normalize the red, green and blue (RGB) values. Color intensity of seed surface areas was measured using ImageJ software^53^. The average RGB value of these 8 seeds was used to represent seed color. Yellow seeds had higher RGB values than brown seeds.

### SSR marker development and genotyping

Simple sequence repeat (SSR) markers were developed by surveying flax genomic sequences using ‘WebSat’ software^54^. Details of these markers are provided in Supp. Table S1. In addition, published SSR markers were also used^20–22,55^. The forward primers used in PCR amplification had on their 5′ side an M13 sequence to enable fluorescence detection and multiplexing (CACGACGTTGTAAAACGAC). SSR amplification and detection was performed as described earlier^55^.

### Development of CAPS (Cleaved Amplified Polymorphic Sequences) markers

Single nucleotide polymorphisms (SNPs) were identified from the alignment of Illumina short reads of G1186/94^56^ to the DNA sequence of scaffold 208 that was obtained from the whole genome assembly of CDC Bethune^24^. Putative SNPs between CDC Bethune and G1186/94 were detected *in silico* (CLC Genomics Workbench 6 software; www.clcbio.com). A SNP was considered putative when present in at least two reads. Such putative SNP targets were subjected to *in silico* restriction digests for all commercially available enzymes; among these, the ones with high read-counts in the assembly file were identified as putative CAPS. LuCAPS_110 was amplified using LuCAPS_110_F (CCTTTATCTCTGCCTCTTCTCC) and LuCAPS_110_R (ACAACCCCAACACAATCTCG) and was digested with *Hae*III. Wild type (CDC Bethune type) produced a unique ~500 bp amplicon whereas *Hae*III restricted the yellow type amplicon into two fragments of ~290 and 220 bp.

### Genetic analyses

Segregation ratio for *d* mutation was assessed using chi-square analysis (Yate’s correction applied) considering an expected 1:1 segregation ratio for yellow: brown seed color in the advanced RIL population^13^. It is assumed that there was no operator bias while advancing RIL population using single seed descent method. Linkage analysis was performed using a minimum LOD 3.0 threshold and a maximum recombination fraction of 40^55^. LOD threshold was determined by 1000 permutations at P < 0.05^57^. QTL analysis was performed using phenotypic-genotypic data in QTL Cartographer v2.5. The composite interval mapping (CIM) method was used (Kosambi function, walk speed 1 cM and ‘forward and backward regression’ method for co-factors, standard model 6 forward and backward regression). QTL identification and percent contribution to the phenotypic variance (*R*^2^) were obtained through this analysis.

### *F3*′*H* (*F3*′5′*H*) gene expression analysis using qRT-PCR

RNA was extracted from flax seed coat at 15 DAF and from petal tissues of CDC Bethune and G1186/94 using a method adapted from Meisel *et al*.^58^. cDNA synthesis was performed using AffinityScript™ QPCR cDNA synthesis kit (Agilent Technologies, Santa Clara, CA). *F3*′5′*H* specific target region primers were used to amplify the third exonic region of the putative gene with an expected amplicon length of 158 bp with the forward primer F3′5′H_q_F (AGCTGATGACGGCTGTTCTT) and the reverse primer F3′5′H_q_R (ATAAACATGCTCCGCCAATC). Elongation factor 1α (EF1α) was used as internal control with forward primer α_F1qEF (TTGGATACAACCCCGACAAAA) and reverse primer α_R1qEF (GGGCCCTTGTACCAGTCAAG)^59^. A 50 μl qRT-PCR reaction had 25 μl SYBR GreenER master mix (@2X), 2 μl cDNA, 1 μl each forward and reverse primers (10 pM stock concentration) and 20 μl double-deionized water. The qRT-PCR reactions were initiated with incubation at 95 °C for 10 min followed by a standard two-step protocol of 95 °C for 15 s; 58 °C for 60 s for a total of 40 cycles. Melting curve analyses beginning with an incubation of 1 min at 60 °C with a gradual increase in temperature of 0.3 °C/15 s to 95 °C for 15 s were performed at the end. qRT-PCR reactions were conducted using a StepOnePlus^TM^ Real Time PCR system and data was collected using StepOne v2.1 software (Applied Biosystems). Gene expression changes were calculated using the 2^−ΔΔ*C*^_*T*_ method^60^ for both seed coat and flower petal samples.

### Cloning of full-length *F3*′5′*H* gene from flax

Genomic DNA was extracted from CDC Bethune and G1186/94 leaves using the CTAB method and the full-length genomic region of the *FLAVONOID 3*′5′ *HYDROXYLASE* (*F3*′5′*H*) gene was amplified using PCR primers: 5′-GCGGATCCGATGTCTACGTCGACGGCCAT-3′ (sense, the *Xba*I site is underlined) and 5′-GCGAATTCACTGGTGGCTTGGTTGGTTCC-3′ (antisense, the *Eco*RI site is underlined), gel purified and cloned into TA cloning vector (ThermoFisher Scientific, Waltham, MA).

### Amino acid alignment and phylogenetic tree construction

Amino acid sequences of F3′H and F3′5′H were taken from BRENDA (http://www.brenda-enzymes.org), Seitz *et al*.^31,32^ and NCBI. Sequences were aligned using the complete alignment option (allowing gapped alignment) of Clustal2X (https://sif.info-ufr.univ-montp2.fr/?q=content/clustal2x). This alignment was used to identify conserved domains and to check amino acid divergence at position 5, 8 and 10 of the substrate recognition site 6 (SRS 6) at the C-terminal region of the proteins^32^. The phylogenetic tree was constructed using “One Click” mode of the phylogeny PhyML application (www.phylogeny.lirmm.fr/phylo_cgi/simple_phylogeny.cgi) and the tree was visualized using TreeDyn (www.phylogeny.fr).

### Phytochemistry

Analyses of phenolic compounds were performed as described before^61^. Briefly, all analyses were done in triplicate; flavonoids were extracted from lyophilized seed tissue samples with aqueous (70%) acetone (plant matter: solvent ration 1:5 w/v). Total PA content in catechin equivalent was measured using a dimethylaminocinnamaldehyde (DMACA) assay^62^. Hydrolysis of flavonoid glycosides were done with 2 N HCl for 1 h at 95 °C. Resultant aglycones were extracted with ethyl acetate and subjected to HPLC analysis. Concentration of bound PA was established using butanol-HCl (5%) assay^27^ and expressed as delphinidin or pelargonidin equivalents.

## Electronic supplementary material

Supplementary Information
