# Publisher Correction: QTL mapping and molecular characterization of the classical *D* locus controlling seed and flower color in *Linum usitatissimum* (flax)

**DOI:** 10.1038/s41598-018-22651-9

**Published:** 2018-03-12

**Authors:** Gurudatt Pavagada Sudarshan, Manoj Kulkarni, Leonid Akhov, Paula Ashe, Hamid Shaterian, Sylvie Cloutier, Gordon Rowland, Yangdou Wei, Gopalan Selvaraj

**Affiliations:** 1 National Research Council of Canada, 110 Gymnasium Place, Saskatoon, SK S7N 0W9 Canada; 2 Agriculture and Agri-Food Canada, 960 Carling Avenue, Ottawa, ON K1A 0C6 Canada; 3 Crop Development Centre, Department of Plant Science, University of Saskatchewan, Agriculture Building, 51 Campus Drive, Saskatoon, SK S7N 5A8 Canada; 4 Department of Biology, University of Saskatchewan, 112 Science Place, Saskatoon, SK S7N 5E2 Canada; 5 Present Address: Bayer CropScience, Crop Analytics Morrisville, TECHIII 407 Davis Drive, Morrisville, NC 27560 USA

Correction to: *Scientific Reports* 10.1038/s41598-017-11565-7, published online 16 November 2017

Due to a technical error in the publication of the original version of this Article, Gurudatt Pavagada Sudarshan, Manoj Kulkarni, and Leonid Akhov—who were originally named as equally contributing authors—were not identified as such. This has now been corrected in the PDF and HTML versions of the paper.

