# Supplementary material for: QTL mapping and molecular characterization of the classical D locus controlling seed and flower color in Linum usitatissimum (flax)
Source: Sci Rep. 2017 Nov 16;7:15751. doi: 10.1038/s41598-017-11565-7 (PMC5691222; doi:10.1038/s41598-017-11565-7)
Supplement: Supplementary file 1 — Supplementary Information [file 41598_2017_11565_MOESM1_ESM.pdf]

Supplementary Information

QTL mapping and molecular characterization of the classical *D* locus controlling seed and flower color in *Linum usitatissimum* (flax)

Gurudatt Pavagada Sudarshan , Manoj Kulkarni, Leonid Akhov, Paula Ashe, Hamid Shaterian, Sylvie Cloutier, Gordon Rowland, Yangdou Wei, Gopalan Selvaraj*

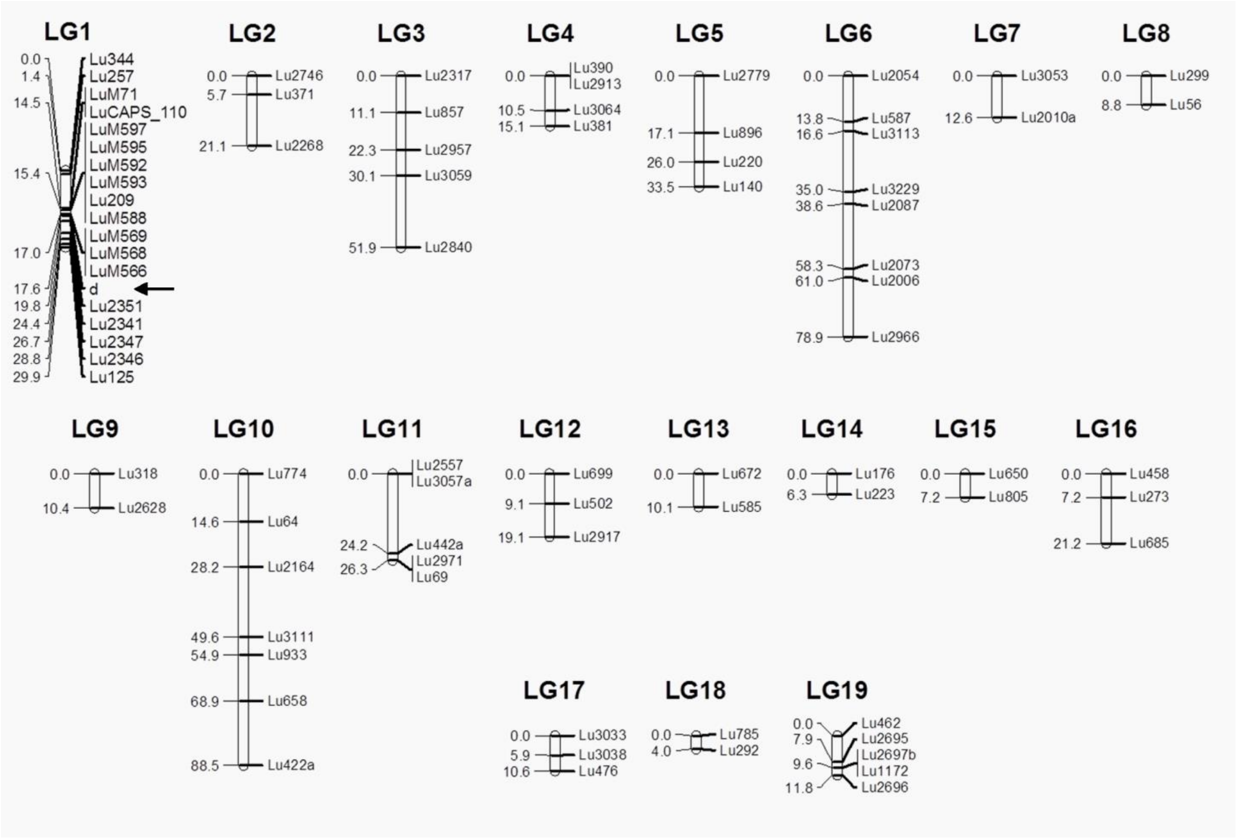

Supplementary Fig. S1. Genetic linkage map of CDC Bethune X G1186/94 mapping population using SSR (EST and genomic) and CAPS markers; LG1 was focused upon after initial association of the *D* locus with LG1. The recessive d allele is marked with an arrow.

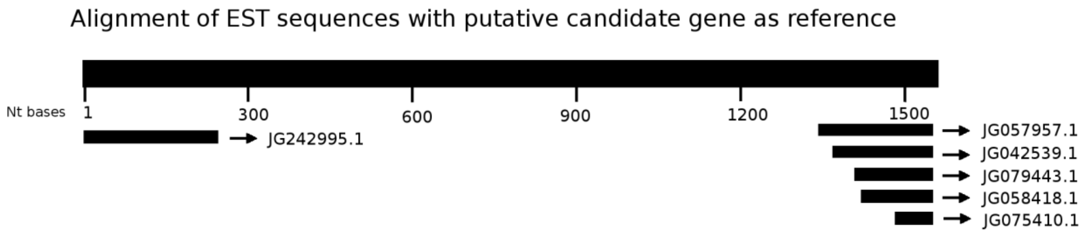

Supplementary Fig. S2. EST sequences found in the NCBI *Linum* EST database using the putative candidate gene F3’5’H as a query (*Lus10021620* in the flax genome). JG242995.1 is 286 bp; JG057957.1 is 404 bp, and the ESTs shown underneath it are nested sequences that are identical to the corresponding stretch in JG057957.1.

```
>Lus10021620
ATGTCTACGTCGACGGCCATCATGTGCGACGGCATTATCATCGCCACCGCCATCTACCTCCTTCTCAGCCTCATCCACCTCCTCCGCCAACGTGGCAGAA
AGCCTCTCCTACCGGGCCCCACTCCGTGGCCGATTGTGGGGAACCTTCTCCACTTGGGTCCAATGCCCCACCACACTATAGCCGCCCTGGCTAGGAGGTA
CGGACCCCTAATATACCTCAGGCTAGGGTACGTGGACGTGGTGGTGGCCGCCTCGGCCTCGGTGGCTGCTCAGTTTTTGAAGCACAACGACTCCAACTTC
TCCAATCGGCCTCAAACTTCTGGTGGGAAGTACATGGCTTACGGCTTCCAGGACATGGTGTTCGCCCCCTACGGCCCACGTTGGAAGCTTCTCCGGAAAG
TCAGCGCCGTCCATCTCTTCTCCGGCAAGGCCTTGGATAATTTTAGACACGTCCGACAGGTATATACATTGTTAATTGTTAGTTCTGTTATTTCAAGCGT
AACTTCATGACAACATTTTTTTTTTTGCATGTCCGTGAAAATTCGAAACAAAAATTCAATAAGTTACATTACTGATGACTAGACTTTTTCTGATCTAATA
GTGATGAAATTTATAAAATTTATACAGAGTATTTTATATAAATTGACTTTTAAACTTAAATTTCATTCTTCAAATTATGCTATAGTTTCGCTATTTGATT
ACTACATTAATGTTCATTCATTTTCATACAAATGTAAATTATCAAACAGAGAGAAGTGACGAGTCTGACTCGGGCGCTAGCGGGTTCTGGCGGCGCGACA
GTGGACCTGGGCTTACTCATAACTTTCTGCTCCACAAACGCGTTGGGGAAAGCGTTAGTTGGGCGTGTAGGTGGGAAACAATGTGGGGGGGACACCCCCG
GCGGTGTCGACCCTAGGGCGGAAAAGTTCAAGACCATGGTGGTGGAGCTTATGACCTTGGCCGGAATTTTCAACATCGGCGACTTCATCCCGGCCGTGGA
ATGGCTAGACTTACAGGGCATTGCTGCTAGGATGAAGAGTGTCCACAACAGGTTCGACTCCTTCTTGAATCAAATACTTGTGGAATACAGGAAGACCGCC
CGCGAGGGAGAAAACGTGGACTTTTTGAGTTCGTTGATGTCGTCAAGAATTGCGGACGGCCCCGATGGAGTGGAGCGTCAGATCACTGACACTGAAATCA
AAGCTTTGCTTCTCGTACGTGATCTCGATCCTCTATTCTTTATTATGATTTAAATTATAATTATGTGCTTTAACTAACTCTTATGCATATATTGTCGAAA
AAAAACTTATGCATATTATTATCACAGAATATGTTCAGTGCCGGAACCGACACGTCATCAAGTACGGTGGAATGGGCCGTGGCGGAGCTAATCCGTCACC
CGAAAGTCCTGACCCAACTCCAAACCGAGCTGGACACCGTAGCGGGCCGAGACCGTCTGGTCAACGAACTTGACATCCCTAACCTCCCATACTTAAACGC
CGTCGTAAAGGAGATCTTCCGTTTACATCCACCAACTCCTCTTTCTCTCCCCCGGATGGCCGCCGAAAGCTGCGAGATCAACGGCCTCCATATCCCGAAA
GGTGCAACACTCTTGGTCAACATATGGGCCATAGGCCGCGATCCGGATGTGTGGTCTGACCCGTTGAGGTTCGACCCGGGCAGGTTCTTGCCCGGTGGGG
AGAAGCCCGGAGTGGGTGTGAAGGGGAGCGACTTCGAGCTGATTCCATTCGGGGCGGGTAGGAGGATCTGCTCGGGTATGAGCTTGGGGCTCCGGACGGT
TCAGCTGATGACGGCTGTTCTTGCCCATGGGTTTGATTGGGAGCTTAAAGACGGAGTCTCGGCGGAGGAGCTGAATATGGATGAGGTTTTCGGGATTTCG
TTACAACGGGCTGTGCCGCTGGTCGTGCGGCCAAAAGCACGATTGGCGGAGCATGTTTATCGAGGGTGAAGGGAACCAACCAAGCCACCAG
```

Green – Introns; Yellow – amplification primers ; Turquoise- qPCR amplification region, whose primers are indicated in Methods.

Supplementary Fig. S3. The sequence of the candidate gene for the *D* locus found in Scaffold 208 sequence corresponding to the mapped position, and annotated as *FLAVONOID 3' HYDROXYLASE* (*F3'H*).

```
Lus10021620        ATGTCTACGTCGACGGCCATCATGTGCGACGGCATTATCATCGCCACCGCCATCTACCTC
F3'H_G1186/94      ATGTCTACGTCGACGGCCATCATGTGCGACGGCATTATCATCGCCACCGCCATCTACCTC
                   ************************************************************

Lus10021620        CTTCTCAGCCTCATCCACCTCCTCCGCCAACGTGGCAGAAAGCCTCTCCTACCGGGCCCC
F3'H_G1186/94      CTTCTCAGCCTCATCCACCTCCTCCGCCAACGTGGCAGAAAGCCTCTCCTACCGGGCCCC
                   ************************************************************

Lus10021620        ACTCCGTGGCCGATTGTGGGGAACCTTCTCCACTTGGGTCCAATGCCCCACCACACTATA
F3'H_G1186/94      ACTCCGTGGCCGATTGTGGGGAACCTTCTCCACTTGGGTCCAATGCCCCACCACACTATA
                   ************************************************************

Lus10021620        GCCGCCCTGGCTAGGAGGTACGGACCCCTAATATACCTCAGGCTAGGGTACGTGGACGTG
F3'H_G1186/94      GCCGCCCTGGCTAGGAGGTACGGACCCCTAATATACCTCAGGCTAGGGTACGTGGACGTG
                   ************************************************************

Lus10021620        GTGGTGGCCGCCTCGGCCTCGGTGGCTGCTCAGTTTTTGAAGCACAACGACTCCAACTTC
F3'H_G1186/94      GTGGTGGCCGCCTCGGCCTCGGTGGCTGCTCAGTTTTTGAAGCACAACGACTCCAACTTC
                   ************************************************************


Lus10021620        TCCAATCGGCCTCAAACTTCTGGTGGGAAGTACATGGCTTACGGCTTCCAGGACATGGTG
F3'H_G1186/94      TCCAATCGGCCTCAAACTTCTGGTGGGAAGTACATGGCTTACGGCTTCCAGGACATGGTG
                   ************************************************************

Lus10021620        TTCGCCCCCTACGGCCCACGTTGGAAGCTTCTCCGGAAAGTCAGCGCCGTCCATCTCTTC
F3'H_G1186/94      TTCGCCCCCTACGGCCCACGTTGGAAGCTTCTCCGGAAAGTCAGCGCCGTCCATCTCTTC
                   ************************************************************

Lus10021620        TCCGGCAAGGCCTTGGATAATTTTAGACACGTCCGACAGGTATATACATTGTTAATTGTT
F3'H_G1186/94      TCCGGCAAGGCCTTGGATAATTTTAGACACGTCCGACAGGTATATACATTGTTAATTGTT
                   ************************************************************

Lus10021620        AGTTCTGTTATTTCAAGCGTAACTTCATGACAACATTTTTTTTTTTGCATGTCCGTGAAA
F3'H_G1186/94      AGTTCTGTTATTTCAAGCGTAACTTCATGACAACATTTTTTTTTTTGCATGTCCGTGAAA
                   ************************************************************

Lus10021620        ATTCGAAACAAAAATTCAATAAGTTACATTACTGATGACTAGACTTTTTCTGATCTAATA
F3'H_G1186/94      ATTCGAAACAAAAATTCAATAAGTTACATTACTGATGACTAGACTTTTTCTGATCTAATA
                   ************************************************************

Lus10021620        GTGATGAAATTTATAAAATTTATACAGAGTATTTTATATAAATTGACTTTTAAACTTAAA
F3'H_G1186/94      GTGATGAAATTTATAAAATTTATACAGAGTATTTTATATAAATTGACTTTTAAACTTAAA
                   ************************************************************

Lus10021620        TTTCATTCTTCAAATTATGCTATAGTTTCGCTATTTGATTACTACATTAATGTTCATTCA
F3'H_G1186/94      TTTCATTCTTCAAATTATGCTATAGTTTCGCTATTTGATTACTACATTAATGTTCATTCA
                   ************************************************************

Lus10021620        TTTTCATACAAATGTAAATTATCAAACAGAGAGAAGTGACGAGTCTGACTCGGGCGCTAG
F3'H_G1186/94      TTTTCATACAAATGTAAATTATCAAACAGAGAGAAGTGACGAGTCTGACTCGGGCGCTAG
                   ************************************************************

Lus10021620        CGGGTTCTGGCGGCGCGACAGTGGACCTGGGCTTACTCATAACTTTCTGCTCCACAAACG
F3'H_G1186/94      CGGGTTCTGGCGGCGCGACAGTGGACCTGGGCTTACTCATAACTTTCTGCTCCACAAACG
                   ************************************************************

Lus10021620        CGTTGGGGAAAGCGTTAGTTGGGCGTGTAGGTGGGAAACAATGTGGGGGGGACACCCCCG
F3'H_G1186/94      CGTTGGGGAAAGCGTTAGTTGGGCGTGTAGGTGGGAAACAATGTGGGGGGGACACCCCCG
                   ************************************************************

Lus10021620        GCGGTGTCGACCCTAGGGCGGAAAAGTTCAAGACCATGGTGGTGGAGCTTATGACCTTGG
F3'H_G1186/94      GCGGTGTCGACCCTAGGGCGGAAAAGTTCAAGACCATGGTGGTGGAGCTTATGACCTTGG
```

```
                    ************************************************************

Lus10021620         CCGGAATTTTCAACATCGGCGACTTCATCCCGGCCGTGGAATGGCTAGACTTACAGGGCA
F3'H_G1186/94       CCGGAATTTTCAACATCGGCGACTTCATCCCGGCCGTGGAATGGCTAGACTTACAGGGCA
                    ************************************************************

Lus10021620         TTGCTGCTAGGATGAAGAGTGTCCACAACAGGTTCGACTCCTTCTTGAATCAAATACTTG
F3'H_G1186/94       TTGCTGCTAGGATGAAGAGTGTCCACAACAGGTTCGACTCCTTCTTGAATCAAATACTTG
                    ************************************************************

Lus10021620         TGGAATACAGGAAGACCGCCCGCGAGGGAGAAAACGTGGACTTTTTGAGTTCGTTGATGT
F3'H_G1186/94       TGGAA-ACAGGAAGACCGCCCGCGAGGGAGAAAACGTGGACTTTTTGAGTTCGTTGATGT
                    ***** ******************************************************

Lus10021620         CGTCAAGAATTGCGGACGGCCCCGATGGAGTGGAGCGTCAGATCACTGACACTGAAATCA
F3'H_G1186/94       CGTCAAGAATTGCGGACGGCCCCGATGGAGTGGAGCGTCAGATCACTGACACTGAAATCA
                    ************************************************************

Lus10021620         AAGCTTTGCTTCTCGTACGTGATCTCGATCCTCTATTCTTTATTATGATTTAAATTATAA
F3'H_G1186/94       AAGCTTTGCTTCTCGTACGTGATCTCGATCCTCTATTCTTTATTATGATTTAAATTATAA
                    ************************************************************

Lus10021620         TTATGTGCTTTAACTAACTCTTATGCATATATTGTCGAAAAAAAACTTATGCATATTATT
F3'H_G1186/94       TTATGTGCTTTAACTAACTCTTATGCATATATTGTCGAAAAAAAACTTATGCATATTATT
                    ************************************************************

Lus10021620         ATCACAGAATATGTTCAGTGCCGGAACCGACACGTCATCAAGTACGGTGGAATGGGCCGT
F3'H_G1186/94       ATCACAGAATATGTTCAGTGCCGGAACCGACACGTCATCAAGTACGGTGGAATGGGCCGT
                    ************************************************************

Lus10021620         GGCGGAGCTAATCCGTCACCCGAAAGTCCTGACCCAACTCCAAACCGAGCTGGACACCGT
F3'H_G1186/94       GGCGGAGCTAATCCGTCACCCGAAAGTCCTGACCCAACTCCAAACCGAGCTGGACACCGT
                    ************************************************************

Lus10021620         AGCGGGCCGAGACCGTCTGGTCAACGAACTTGACATCCCTAACCTCCCATACTTAAACGC
F3'H_G1186/94       AGCGGGCCGAGACCGTCTGGTCAACGAACTTGACATCCCTAACCTCCCATACTTAAACGC
                    ************************************************************

Lus10021620         CGTCGTAAAGGAGATCTTCCGTTTACATCCACCAACTCCTCTTTCTCTCCCCCGGATGGC
F3'H_G1186/94       CGTCGTAAAGGAGATCTTCCGTTTACATCCACCAACTCCTCTTTCTCTCCCCCGGATGGC
                    ************************************************************

Lus10021620         CGCCGAAAGCTGCGAGATCAACGGCCTCCATATCCCGAAAGGTGCAACACTCTTGGTCAA
F3'H_G1186/94       CGCCGAAAGCTGCGAGATCAACGGCCTCCATATCCCGAAAGGTGCAACACTCTTGGTCAA
                    ************************************************************

Lus10021620         CATATGGGCCATAGGCCGCGATCCGGATGTGTGGTCTGACCCGTTGAGGTTCGACCCGGG
F3'H_G1186/94       CATATGGGCCATAGGCCGCGATCCGGATGTGTGGTCTGACCCGTTGAGGTTCGACCCGGG
                    ************************************************************

Lus10021620         CAGGTTCTTGCCCGGTGGGGAGAAGCCCGGAGTGGGTGTGAAGGGGAGCGACTTCGAGCT
F3'H_G1186/94       CAGGTTCTTGCCCGGTGGGGAGAAGCCCGGAGTGGGTGTGAAGGGGAGCGACTTCGAGCT
                    ************************************************************

Lus10021620         GATTCCATTCGGGGCGGGTAGGAGGATCTGCTCGGGTATGAGCTTGGGGCTCCGGACGGT
F3'H_G1186/94       GATTCCATTCGGGGCGGGTAGGAGGATCTGCTCGGGTATGAGCTTGGGGCTCCGGACGGT
                    ************************************************************

Lus10021620         TCAGCTGATGACGGCTGTTCTTGCCCATGGGTTTGATTGGGAGCTTAAAGACGGAGTCTC
F3'H_G1186/94       TCAGCTGATGACGGCTGTTCTTGCCCATGGGTTTGATTGGGAGCTTAAAGACGGAGTCTC
                    ************************************************************

Lus10021620         GGCGGAGGAGCTGAATATGGATGAGGTTTTCGGGATTTCGTTACAACGGGCTGTGCCGCT
```

```
F3'H_G1186/94      GGCGGAGGAGCTGAATATGGATGAGGTTTTCGGGATTTCGTTACAACGGGCTGTGCCGCT
                   ************************************************************

Lus10021620        GGTCGTGCGGCCAAAAGCACGATTGGCGGAGCATGTTTATCGAGGGTGA
F3'H_G1186/94      GGTCGTGCGGCCAAAAGCACGATTGGCGGAGCATGTTTATCGAGGGTGA
                   *************************************************
```

Supplementary Fig. S4. Genomic sequence alignment showing the single nucleotide deletion mutation in the *d* mutant gene. Upper sequence, CDC Bethune; lower sequence, G1186/94. Starting from the 5' end: The initiation codon is highlighted in green; the first intron is highlighted in brown; deletion of the nucleotide "T" in G1186/94 sequence at position 1086 relative to the initiation codon is indicated with a hyphen highlighted in red, and the resultant premature termination codon TGA downstream of the deletion due to frameshift in the G1186/94 sequence is highlighted in teal; the second intron is highlighted in brown; the termination codon in the CDC Bethune sequence is highlighted in teal. The single nucleotide deletion in G1186/94 has been confirmed by 5 independent PCR reactions followed by sequencing to rule out errors.

```
Lus10021620              MSTSTAIMCDGIIIATAIYLLLSLIHLLRQRGRKPLLPGPTPWPIVGNLLHLGPMPHHTI      60
G1186/94_F3'5H_trun      MSTSTAIMCDGIIIATAIYLLLSLIHLLRQRGRKPLLPGPTPWPIVGNLLHLGPMPHHTI      60
                         ************************************************************

Lus10021620              AALARRYGPLIYLRLGYVDVVVAASASVAAQFLKHNDSNFSNRPQTSGGKYMAYGFQDMV      120
G1186/94_F3'5H_trun      AALARRYGPLIYLRLGYVDVVVAASASVAAQFLKHNDSNFSNRPQTSGGKYMAYGFQDMV      120
                         ************************************************************

Lus10021620              FAPYGPRWKLLRKVSAVHLFSGKALDNFRHVRQREVTSLTRALAGSGGATVDLGLLITFC      180
G1186/94_F3'5H_trun      FAPYGPRWKLLRKVSAVHLFSGKALDNFRHVRQREVTSLTRALAGSGGATVDLGLLITFC      180
                         ************************************************************

Lus10021620              STNALGKALVGRVGGKQCGGDTPGGVDPRAEKFKTMVVELMTLAGIFNIGDFIPAVEWLD      240
G1186/94_F3'5H_trun      STNALGKALVGRVGGKQCGGDTPGGVDPRAEKFKTMVVELMTLAGIFNIGDFIPAVEWLD      240
                         ************************************************************

Lus10021620              LQGIAARMKSVHNRFDSFLNQILVEYRKTAREGENVDFLSSLMSSRIADGPDGVERQITD      300
G1186/94_F3'5H_trun      LQGIAARMKSVHNRFDSFLNQILVETGRPPAREKTWTFS∞---------------------    278
                         *************************

Lus10021620              TEIKALLLNMFSAGTDTSSSTVEWAVAELIRHPKVLTQLQTELDTVAGRDRLVNELDIPN      360
G1186/94_F3'5H_trun      ------------------------------------------------------------      278

Lus10021620              LPYLNAVVKEIFRLHPPTPLSLPRMAAESCEINGLHIPKGATLLVNIWAIGRDPDVWSDP      420
G1186/94_F3'5H_trun      ------------------------------------------------------------      278

Lus10021620              LRFDPGRFLPGGEKPGVGVKGSDFELIPFGAGRRICSGMSLGLRTVQLMTAVLAHGFDWE      480
G1186/94_F3'5H_trun      ------------------------------------------------------------      278

Lus10021620              LKDGVSAEELNMDEVFGISLQRAVPLVVRPKARLAEHVYRG∞        521
G1186/94_F3'5H_trun      ------------------------------------------        278
```

Supplementary Fig. S5. Alignment of the CDC Bethune and G1186/94 sequences corresponding to the *D* locus gene and the *d* allele. The frameshift in the latter from G1186/94 is indicated by grey highlight and the resultant premature termination by an omega symbol. The CDC Bethune sequence has 521 amino acids whereas the G1186/94 sequence has been truncated to 278 amino acids, of which the 14 amino acids in grey highlight at the carboxy region are variants due to the frameshift. The amino acids at positions 5, 8 and 10 of the Substrate Recognition Site 6 (SRS6) in CDC Bethune sequence are highlighted in yellow, turquoise and red, respectively [31,32].

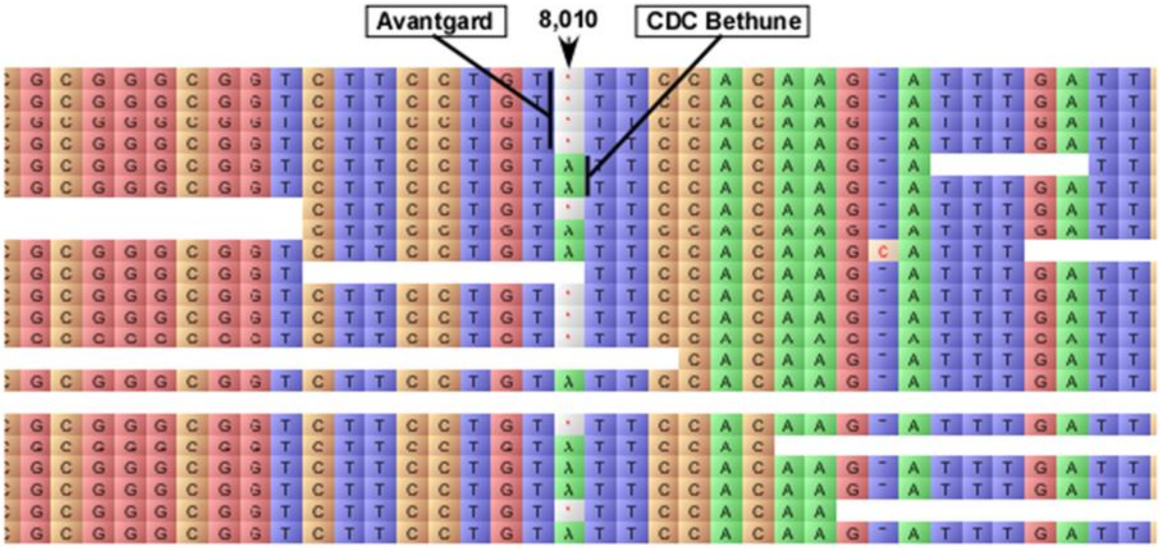

Supplementary Fig. S6. Avantgard, yellow-seeded *d* mutant parent of G1186/94, sequenced independently also shows the single nucleotide deletion found in G1186/94. The sequences shown here are the complementary strand with reference to the sequence in Suppl. Fig. 3. Note the presence of A in CDC Bethune short sequence reads and its absence in multiple short sequence reads from Avantgard. The sequences below the marked ones are of short reads from CDC Bethune where an A is present at position 8010 and of Avantgard where the A is absent. The position 8010 refers to the location in Scaffold 208 [24].

Supplementary Table S1. Simple sequence repeat markers, the forward and reverse primers and amplicon length polymorphisms.

	Marker name	Fragment length (bp)		Primer name	Sequence (5' to 3')	Tm (°C)	Primer length (mer)
		CDC Bethune	G1186/94				
1	LuM3	204	207	LuMF3	ACGGAAGGTTTAGTGTCGAGAA	58	22
				LuMR3	ATGGGAAGAAGGTAAAGCCAAT	58	22
2	LuM18	211	229	LuMF18	TTGCATAGACAAATGGCTCATC	58	22
				LuMR18	ACCAACAACGCAAAAGCATAG	58	21
3	LuM47	233	231	LuMF47	CAAATCAGAATGTGCGTGTGTA	58	22
				LuMR47	GGACGTTATGGTCTCTGCTCTC	58	22
4	LuM56	247	244	LuMF56	AATAGATGCTAGAGTGCTCGCC	58	22
				LuMR56	TCGTCCATAAGCTGGAAATCTT	58	22
5	LuM63	239	245	LuMF63	ACAGCTAGGGGTAGGCCAGT	58	20
				LuMR63	CCCACAACACACACAAATAACC	58	22
6	LuM67	132	133	LuMF67	ATAGGATAGGACATGACGAGCC	58	22
				LuMR67	TGCTGAGAAGGTGAAGACTGAA	58	22
7	LuM68	298	300	LuMF68	TAAATCAGTCAGGTTCGGTTTG	57	22
				LuMR68	CGCATCAGAATCCATCGTATAA	58	22
8	LuM71	369	367	LuMF71	AGAAAATCGAAAGATGAAGGGG	59	22
				LuMR71	CTACTCTTCCCACGTTGACCA	58	21
9	LuM73	229	224	LuMF73	GGTCTAACGGAATGCCTATCAG	58	22
				LuMR73	CATGTCTTCCCCTTCTCACTTC	58	22
10	LuM82	295	298	LuMF82	CGCATCAGAATCCATCGTATAA	58	22
				LuMR82	TAAATCAGTCAGGTTCGGTTTG	57	22
11	LuM84	231	250	LuMF84	CGACAGTTCGTAGGAGAGAAATAA	57	24
				LuMR84	TTGAGTTGATATGTTCCGCTGT	58	22
12	LuM88	146	140	LuMF88	ACTGCTTTGAATCGGAGAGAAG	58	22
				LuMR88	GGGGATGAAGGTCTACCATGT	58	21
13	LuM93	341	313	LuMF93	ATTTACTAGCAGCAGGTTGGGA	58	22
				LuMR93	GGAAAACATACGATAGCCAAGC	58	22
14	LuM101	196	184	LuMF101	ATAGGTAGTGCCGTTGGGTTT	58	21
				LuMR101	AACAAAGGGTAGCCATGAAGAA	58	22
15	LuM106	174	162	LuMF106	AGAAGAGGGCATCCAACAAAG	59	21
				LuMR106	AACAAAGGGTAGCCATGAAGAA	58	22
16	LuM109	255	313	LuMF109	CATTTTGTGGAAGGACAACAAG	58	22
				LuMR109	ACAATGATAGGAACAGCACGTC	57	22
17	LuM126	346	343	LuMF126	GCTTCTTCTTCTTCACAACATCAC	58	24
				LuMR126	CCGGAACCCCTTACAAAGAT	58	20
18	LuM132	231	240, 241	LuMF132	AGAAGAGAGGATGGGGTTGAAG	59	22
				LuMR132	ATGTTTATTAGTGGGTGGTCGG	58	22
19	LuM134	174	162	LuMF134	AGAAGAGGGCATCCAACAAAG	59	21
				LuMR134	AACAAAGGGTAGCCATGAAGAA	58	22
20	LuM137	212	211	LuMF137	AACCAGAAACAGAAACAGAGGC	58	22
				LuMR137	ATACCTGGATTGGAGTTGGAGA	58	22

21	LuM140	255	252	LuMF140	CAAACAAGAACCCTAAAACCCA	58	22
				LuMR140	TTGGATCACTAACGCTGCAAT	59	21
22	LuM147	225	226	LuMF147	TGGAGGAGGAGTGGTAAGAAAA	58	22
				LuMR147	ATAGGATAGGACATGACGAGCC	58	22
23	LuM149	248	304	LuMF149	ACAATGATAGGAACAGCACGTC	57	22
				LuMR149	CATTTTGTGGAAGGACAACAAG	58	22
24	LuM152	276	282	LuMF152	GACTGTAGCAAACACCATGCAG	59	22
				LuMR152	CTCCTAAACCCACTCACCAAAG	58	22
25	LuM159	321	251	LuMF159	TCAACATCCTTTTGTGTCCAAC	58	22
				LuMR159	TTCTGCTGTTTCGTTAGCAAAG	58	22
26	LuM162	127	151	LuMF162	CCATCTCCTTCATTCTTACCTCC	58	23
				LuMR162	TCAATAAACAACAGCGGAACTG	58	22
27	LuM167	162	138	LuMF167	TGCGTTTCAGTCTCTTTTGTGT	58	22
				LuMR167	CTTTGTCAGGCTCCTTCTTTTG	58	22
28	LuM170	255	313	LuMF170	CATTTTGTGGAAGGACAACAAG	58	22
				LuMR170	ACAATGATAGGAACAGCACGTC	57	22
29	LuM171	191, 194	179, 182	LuMF171	AAGAGGAACAAAGGGTAGCCA	58	21
				LuMR171	GTAGTGCCGTTGGGTTTGAG	59	20
30	LuM172	288	279	LuMF172	AAAGCGATGGAGAAATTAGGTG	58	22
				LuMR172	ACAGTGCGTAGGGGAGAAATAA	58	22
31	LuM173	288	278	LuMF173	AAAGCGATGGAGAAATTAGGTG	58	22
				LuMR173	ACAGTGCGTAGGGGAGAAATAA	58	22
32	LuM177	282	273	LuMF177	ACAGTGCGTAGGGGAGAAATAA	58	22
				LuMR177	AAAGCGATGGAGAAATTAGGTG	58	22
33	LuM185	287	278, 279	LuMF185	AAAGCGATGGAGAAATTAGGTG	58	22
				LuMR185	ACAGTGCGTAGGGGAGAAATAA	58	22
34	LuM186	238	232	LuMF186	ATGGAGTGTATGACAGCAGACG	58	22
				LuMR186	CAAAATGTTCCTTCCTTCCTTG	58	22
35	LuM188	407	401	LuMF188	GCTTTATGGCAAGCTCTATCGT	58	22
				LuMR188	GCCAGATTTATGCTCGTGATCT	59	22
36	LuM193	129	127	LuMF193	TTATGTGTGGGAATTGGACACT	57	22
				LuMR193	GGCCAACTAACTCCTGAAACTC	57	22
37	LuM221	146	148	LuMF221	CAGTGCGATCAATAGAGTTGCT	58	22
				LuMR221	AAAGCATGGAGATAGGGTGAGA	58	22
38	LuM231	145	148	LuMF231	CAGTGCGATCAATAGAGTTGCT	58	22
				LuMR231	AAAGCATGGAGATAGGGTGAGA	58	22
39	LuM241	145	148	LuMF241	CAGTGCGATCAATAGAGTTGCT	58	22
				LuMR241	AAAGCATGGAGATAGGGTGAGA	58	22
40	LuM246	129	104	LuMF246	TTATGTGTGGGAATTGGACACT	57	22
				LuMR246	GGCCAACTAACTCCTGAAACTC	57	22
41	LuM248	133	130, 131	LuMF248	ATCGAGGAGATTTGTTGCC	56	19
				LuMR248	CAAGCCTAGCATCTGAAGTTTT	56	22
42	LuM249	113	105	LuMF249	TTATGTGTGGGAATTGGACACT	57	22
				LuMR249	GGCCAACTAACTCCTGAAACTC	57	22
43	LuM253	129	127	LuMF253	TTATGTGTGGGAATTGGACACT	57	22
				LuMR253	GGCCAACTAACTCCTGAAACTC	57	22

44	LuM262	130	127	LuMF262	TTATGTGTGGGAATTGGACACT	57	22
				LuMR262	GGCCAACTAACTCCTGAAACTC	57	22
45	LuM263	145	149	LuMF263	CAGTGCGATCAATAGAGTTGCT	58	22
				LuMR263	AAAGCATGGAGATAGGGTGAGA	58	22
46	LuM266	216	222	LuMF266	ATACTTGCTGAGTGCGAAAGC	58	21
				LuMR266	GTCCTAATACTGCCCTCTTCCA	58	22
47	LuM272	230	241	LuMF272	TTCCAGTTCTCACCATTCTCAC	57	22
				LuMR272	TAGAGCCCGAAATCAAAAGAAG	58	22
48	LuM280	215	216	LuMF280	TCTCTCTCTCTGTTTTCTGGGAGT	58	24
				LuMR280	AGATTGAGGAGTTTGGTTGGTG	58	22
49	LuM286	174	170	LuMF286	AGTGGAAAGTGCCATTCTGTTT	58	22
				LuMR286	CGGTGTTAGTAGATGCTTCGGT	59	22
50	LuM322	266	254	LuMF322	CTTTGCTTCCTACTCACCCCTT	59	22
				LuMR322	ACAGAGACAGAACCGCAGTCAT	59	22
51	LuM327	266	254	LuMF327	CTTTGCTTCCTACTCACCCCTT	59	22
				LuMR327	ACAGAGACAGAACCGCAGTCAT	59	22
52	LuM331	266	254	LuMF331	CTTTGCTTCCTACTCACCCCTT	59	22
				LuMR331	ACAGAGACAGAACCGCAGTCAT	59	22
53	LuM333	266	254	LuMF333	CTTTGCTTCCTACTCACCCCTT	59	22
				LuMR333	ACAGAGACAGAACCGCAGTCAT	59	22
54	LuM337	274	262	LuMF337	TTATTCCCCTTTGCTTCCAACT	59	22
				LuMR337	ACAGAGACAGAACCGCAGTCAT	59	22
55	LuM339	271	259	LuMF339	TATTCCCCTTTGCTTCCAACT	58	21
				LuMR339	CAGAGACAGAATCGCAGTCATC	58	22
56	LuM347	272	260	LuMF347	TTATTCCCCTTTGCTTCCTACTC	58	23
				LuMR347	TAGAGACAGAACCGCAGTCATC	58	22
57	LuM358	272	262	LuMF358	TTATTCCCCTTTGCTTCCAACT	59	22
				LuMR358	ACAGAGACAGAACCGCAGTCAT	59	22
58	LuM361	271	259	LuMF361	TATTCCCCTTTGCTTCCAACT	58	21
				LuMR361	CAGAGACAGAATCGCAGTCATC	58	22
59	LuM365	266	254	LuMF365	CTTTGCTTCCTACTCACCCCTT	59	22
				LuMR365	ACAGAGACAGAACCGCAGTCAT	59	22
60	LuM370	275	263	LuMF370	CTTATTCCCCTTTGCTTCCAAC	59	22
				LuMR370	ACAGAGACAGAACCGCAGTCAT	59	22
61	LuM371	219	207	LuMF371	ACCCCTCCACTCCCTTTATTC	59	21
				LuMR371	GGATGACGAGGAAATTGGGTAT	59	22
62	LuM372	274	262	LuMF372	TTATTCCCCTTTGCTTCCAACT	59	22
				LuMR372	ACAGAGACAGAACCGCAGTCAT	59	22
63	LuM374	201	188	LuMF374	AATCCCTCCACTCCCTTTATTC	58	22
				LuMR374	TATACAGCCAAACGCCATTGTA	58	22
64	LuM377	266	254	LuMF377	CTTTGCTTCCTACTCACCCCTT	59	22
				LuMR377	ACAGAGACAGAACCGCAGTCAT	59	22
65	LuM391	264	266	LuMF391	GTTGGCCTGTTTGGTTAGGTT	58	21
				LuMR391	CGGGGAGGTATAGATTGTTCTG	58	22
66	LuM404	184	186	LuMF404	GTGCGATCAATAGAGTTGCTTG	58	22
				LuMR404	ACACAGAATCGAAACACAAACG	58	22

67	LuM408	239	234	LuMF408	GGCCAACTAACTCCTGAAACAC	58	22
				LuMR408	GGGAGGCAACCCATGTCTA	59	19
68	LuM413	400	403	LuMF413	CCAAATCAACCCATTAGATGCT	58	22
				LuMR413	ACCGTTGTGTCCTTGTCTTCTT	58	22
69	LuM426	383	385	LuMF426	GGTAGAGTGACCGATGAGTTCC	58	22
				LuMR426	ACAACAACAACCACAACAGGTC	58	22
70	LuM427	332	323	LuMF427	GGGGAGATACGAGTTGATGATT	57	22
				LuMR427	ATGCTTGCAGTCACAGACATTT	58	22
71	LuM429	384	363	LuMF429	AAGGATTTGGTAAAGAGGGGAG	58	22
				LuMR429	AGTCCAAGGGAAGCACAAGTAG	58	22
72	LuM448	400	403	LuMF448	CCAAATCAACCCATTAGATGCT	58	22
				LuMR448	ACCGTTGTGTCCTTGTCTTCTT	58	22
73	LuM457	109	111	LuMF457	GTAAAGCAGTCAAACCCTGGTC	58	22
				LuMR457	TCTTGAAACAGCCAACCCTTAT	58	22
74	LuM468	378	381	LuMF468	TTTGCTCTACCTCTTTGGGTTC	58	22
				LuMR468	TGTGGTCGATACAAGTGAAAGG	58	22
75	LuM566	333	323	LuMF566	TTTTCCTTCACCTAGCTCACTT	60	22
				LuMR566	AAGTTTAGGGGTCCAATCGT	60	20
76	LuM568	289	285	LuMF568	ATCCTCCCTCCGTAGCATAG	60	20
				LuMR568	AGTGTTGAAGAATTGCAGGC	60	20
77	LuM569	289	285	LuMF569	ATCCTCCCTCCGTAGCATAG	60	20
				LuMR569	AGTGTTGAAGAATTGCAGGC	60	20
78	LuM588	346	344	LuMF588	CACACACAAAGATGCCGTTA	60	20
				LuMR588	ATGTGAGATGGGAATGATGG	60	20
79	LuM592	366	368	LuMF592	TTAATTGGTGGGATGGAGAA	60	20
				LuMR592	CTTATCACAAAGCGAGTAGTTACG	60	24
80	LuM595	291	290	LuMF595	AATCCTCTGTTTCTCCCCTG	60	20
				LuMR595	AGAGTGCCATCAGTTTGAGC	60	20
81	LuM597	314	312	LuMF597	TCCGACTATTTCGGGGTTAT	60	20
				LuMR597	TTTTCCTGTGGAGTGTCGAT	60	20

Supplementary Table S2. BLAST results of FGENESH predicted genes encoded on scaffold 208. The gene of interest referred to in the text by homology search against *Arabidopsis thaliana* gene models in TAIR database is highlighted in yellow.

<table>
<tr><th>#</th><th>ID</th><th>TAIR ID</th><th>%ID</th><th>Length</th><th>mismatch</th><th>gap</th><th>qstart</th><th>qend</th><th>sstart</th><th>send</th><th>evalue</th><th>bitscore</th><th>Description</th><th>Exons</th><th>Position</th><th>Protein Size</th><th>Strand</th></tr>
<tr><td>1</td><td>FGENESH:1</td><td>AT1G18650.1</td><td>68.89</td><td>90</td><td>27</td><td>1</td><td>251</td><td>339</td><td>20</td><td>109</td><td>4e-38</td><td>138</td><td>Symbols: PDCB3 | plasmodesmata callose-binding protein 3 | chr1:6419036-6420413 REVERSE</td><td>7 exon (s)</td><td>654 - 4897</td><td>436 aa</td><td>chain -</td></tr>
<tr><td>2</td><td>FGENESH:2</td><td>AT4G19710.2</td><td>50</td><td>30</td><td>10</td><td>2</td><td>74</td><td>103</td><td>206</td><td>230</td><td>0.59</td><td>29.3</td><td>Symbols: AK-HSDH II, AK-HSDH | aspartate kinase-homoserine dehydrogenase ii | chr4:107252</td><td>3 exon (s)</td><td>5224 - 6099</td><td>124 aa</td><td>chain +</td></tr>
<tr><td>3</td><td>FGENESH:3</td><td>AT5G07990.1</td><td>61.69</td><td>509</td><td>186</td><td>4</td><td>5</td><td>512</td><td>8</td><td>508</td><td>0</td><td>656</td><td>Symbols: TT7, CYP75B1, D501 | Cytochrome P450 superfamily protein | chr5:2560437-2562859</td><td>3 exon (s)</td><td>7127 - 9074</td><td>514 aa</td><td>chain -</td></tr>
<tr><td>4</td><td>FGENESH:4</td><td>AT5G11040.1</td><td>26.47</td><td>68</td><td>44</td><td>1</td><td>66</td><td>133</td><td>272</td><td>333</td><td>1.3</td><td>29.3</td><td>Symbols: TRS120, AtTRS120 | TRS120 | chr5:3495332-3500610 FORWARD LENGTH=1186</td><td>5 exon (s)</td><td>10278 - 13980</td><td>178 aa</td><td>chain +</td></tr>
<tr><td>5</td><td>FGENESH:5</td><td>AT1G11100.2</td><td>26.95</td><td>141</td><td>69</td><td>7</td><td>3</td><td>119</td><td>693</td><td>823</td><td>3.1</td><td>27.3</td><td>Symbols: | SNF2 domain-containing protein / helicase domain-containing protein / zinc finger pro</td><td>1 exon (s)</td><td>15246 - 15611</td><td>121 aa</td><td>chain -</td></tr>
<tr><td>6</td><td>FGENESH:6</td><td>AT5G03040.1</td><td>30.77</td><td>39</td><td>27</td><td>0</td><td>88</td><td>126</td><td>207</td><td>245</td><td>0.76</td><td>29.3</td><td>Symbols: iqd2 | IQ-domain 2 | chr5:710380-712406 REVERSE LENGTH=461</td><td>2 exon (s)</td><td>17050 - 17603</td><td>138 aa</td><td>chain -</td></tr>
<tr><td>7</td><td>FGENESH:7</td><td>AT2G21590.1</td><td>29.33</td><td>75</td><td>37</td><td>5</td><td>1</td><td>68</td><td>224</td><td>289</td><td>0.89</td><td>26.9</td><td>Symbols: APL4 | Glucose-1-phosphate adenylyltransferase family protein | chr2:9239362-9242150</td><td>1 exon (s)</td><td>20388 - 20612</td><td>74 aa</td><td>chain -</td></tr>
<tr><td>8</td><td>FGENESH:8</td><td>AT5G07980.1</td><td>37.78</td><td>540</td><td>271</td><td>15</td><td>1</td><td>510</td><td>1</td><td>505</td><td>7e-72</td><td>265</td><td>Symbols: | dentin sialophosphoprotein-related | chr5:2549432-2554669 REVERSE LENGTH=15</td><td>6 exon (s)</td><td>22686 - 27964</td><td>1366 aa</td><td>chain +</td></tr>
<tr><td>9</td><td>FGENESH:9</td><td>AT5G07950.1</td><td>41</td><td>239</td><td>101</td><td>5</td><td>57</td><td>295</td><td>74</td><td>272</td><td>1e-47</td><td>164</td><td>Symbols: | unknown protein; FUNCTIONS IN: molecular_function unknown; INVOLVED IN: bi</td><td>8 exon (s)</td><td>28131 - 30004</td><td>314 aa</td><td>chain -</td></tr>
<tr><td>10</td><td>FGENESH:10</td><td>AT5G07960.1</td><td>55.71</td><td>140</td><td>21</td><td>1</td><td>2</td><td>141</td><td>9</td><td>107</td><td>6e-47</td><td>150</td><td>Symbols: | unknown protein; CONTAINS InterPro DOMAIN/s: Uncharacterised protein family U</td><td>4 exon (s)</td><td>30347 - 32172</td><td>145 aa</td><td>chain +</td></tr>
<tr><td>11</td><td>FGENESH:11</td><td>AT5G39510.1</td><td>74.87</td><td>199</td><td>50</td><td>0</td><td>1</td><td>199</td><td>1</td><td>199</td><td>1e-104</td><td>319</td><td>Symbols: VTI11, ATVTI1A, ATVTI11, ZIG, SGR4, VTI1A, ZIG1 | Vesicle transport v-SNARE f</td><td>11 exon (s)</td><td>33577 - 37672</td><td>637 aa</td><td>chain -</td></tr>
<tr><td>12</td><td>FGENESH:12</td><td>ATMG00810.1</td><td>43.14</td><td>51</td><td>25</td><td>1</td><td>85</td><td>131</td><td>151</td><td>201</td><td>5e-10</td><td>55.8</td><td>Symbols: ORF240B | DNA/RNA polymerases superfamily protein | chrM:227709-228431 REVER</td><td>3 exon (s)</td><td>43051 - 44019</td><td>161 aa</td><td>chain -</td></tr>
<tr><td>13</td><td>FGENESH:13</td><td>AT1G77470.1</td><td>29.17</td><td>72</td><td>44</td><td>2</td><td>37</td><td>101</td><td>45</td><td>116</td><td>0.24</td><td>29.6</td><td>Symbols: RFC3, RFC5 | replication factor C subunit 3 | chr1:29112194-29114323 REVERSE LEN</td><td>2 exon (s)</td><td>46323 - 47364</td><td>108 aa</td><td>chain +</td></tr>
<tr><td>14</td><td>FGENESH:14</td><td>AT5G07920.1</td><td>71.64</td><td>744</td><td>175</td><td>6</td><td>1</td><td>725</td><td>1</td><td>727</td><td>0</td><td>1096</td><td>Symbols: DGK1, ATDGK1 | diacylglycerol kinase1 | chr5:2525197-2528396 REVERSE LENGTH</td><td>7 exon (s)</td><td>48975 - 52821</td><td>726 aa</td><td>chain +</td></tr>
<tr><td>15</td><td>FGENESH:15</td><td>AT5G15490.1</td><td>86.3</td><td>467</td><td>61</td><td>1</td><td>1</td><td>467</td><td>17</td><td>480</td><td>0</td><td>840</td><td>Symbols: | UDP-glucose 6-dehydrogenase family protein | chr5:5027872-5029314 REVERSE LE</td><td>3 exon (s)</td><td>54135 - 56330</td><td>467 aa</td><td>chain +</td></tr>
<tr><td>16</td><td>FGENESH:16</td><td>AT4G13750.1</td><td>58.82</td><td>17</td><td>7</td><td>0</td><td>107</td><td>123</td><td>77</td><td>93</td><td>3.8</td><td>28.1</td><td>Symbols: NOV | Histidine kinase-, DNA gyrase B-, and HSP90-like ATPase family protein | chr4:</td><td>5 exon (s)</td><td>56729 - 58884</td><td>195 aa</td><td>chain -</td></tr>
<tr><td>17</td><td>FGENESH:17</td><td>AT5G61410.1</td><td>75</td><td>304</td><td>33</td><td>5</td><td>2</td><td>303</td><td>4</td><td>266</td><td>1e-152</td><td>434</td><td>Symbols: RPE, EMB2728 | D-ribulose-5-phosphate-3-epimerase | chr5:24684085-24685836 REV</td><td>11 exon (s)</td><td>59314 - 62761</td><td>330 aa</td><td>chain +</td></tr>
<tr><td>18</td><td>FGENESH:18</td><td>AT1G74410.1</td><td>61.21</td><td>214</td><td>73</td><td>3</td><td>38</td><td>245</td><td>12</td><td>221</td><td>1e-82</td><td>250</td><td>Symbols: | RING/U-box superfamily protein | chr1:27965723-27967681 FORWARD LENGTH=</td><td>5 exon (s)</td><td>63204 - 64267</td><td>245 aa</td><td>chain -</td></tr>
<tr><td>19</td><td>FGENESH:19</td><td>AT1G21280.1</td><td>24.43</td><td>131</td><td>83</td><td>4</td><td>7</td><td>126</td><td>100</td><td>225</td><td>6e-06</td><td>47.4</td><td>Symbols: | CONTAINS InterPro DOMAIN/s: Retrotransposon gag protein (InterPro:IPR005162);</td><td>2 exon (s)</td><td>67256 - 68800</td><td>498 aa</td><td>chain -</td></tr>
<tr><td>20</td><td>FGENESH:20</td><td>AT5G61380.1</td><td>46.19</td><td>236</td><td>89</td><td>9</td><td>88</td><td>305</td><td>379</td><td>594</td><td>2e-46</td><td>167</td><td>Symbols: TOC1, APRR1, PRR1, AtTOC1 | CCT motif -containing response regulator protein | ch</td><td>2 exon (s)</td><td>69785 - 70800</td><td>323 aa</td><td>chain -</td></tr>
<tr><td>21</td><td>FGENESH:21</td><td>AT4G23160.1</td><td>41.3</td><td>552</td><td>310</td><td>6</td><td>67</td><td>608</td><td>51</td><td>598</td><td>2e-144</td><td>454</td><td>Symbols: CRK8 | cysteine-rich RLK (RECEPTOR-like protein kinase) 8 | chr4:12129485-121340</td><td>1 exon (s)</td><td>73684 - 75534</td><td>616 aa</td><td>chain +</td></tr>
<tr><td>22</td><td>FGENESH:22</td><td>AT5G61380.1</td><td>71.86</td><td>199</td><td>56</td><td>0</td><td>20</td><td>218</td><td>9</td><td>207</td><td>3e-96</td><td>297</td><td>Symbols: TOC1, APRR1, PRR1, AtTOC1 | CCT motif -containing response regulator protein | ch</td><td>5 exon (s)</td><td>75713 - 77540</td><td>251 aa</td><td>chain -</td></tr>
<tr><td>23</td><td>FGENESH:23</td><td>AT4G23160.1</td><td>39.81</td><td>309</td><td>132</td><td>2</td><td>338</td><td>592</td><td>75</td><td>383</td><td>1e-59</td><td>221</td><td>Symbols: CRK8 | cysteine-rich RLK (RECEPTOR-like protein kinase) 8 | chr4:12129485-121340</td><td>12 exon (s)</td><td>78212 - 85537</td><td>904 aa</td><td>chain -</td></tr>
<tr><td>24</td><td>FGENESH:24</td><td>AT5G61370.1</td><td>60.32</td><td>436</td><td>171</td><td>1</td><td>17</td><td>450</td><td>15</td><td>450</td><td>0</td><td>565</td><td>Symbols: | Pentatricopeptide repeat (PPR) superfamily protein | chr5:24672008-24673471 REVE</td><td>7 exon (s)</td><td>86428 - 92804</td><td>999 aa</td><td>chain +</td></tr>
<tr><td>25</td><td>FGENESH:25</td><td>AT5G58230.1</td><td>41.38</td><td>29</td><td>16</td><td>1</td><td>71</td><td>99</td><td>24</td><td>51</td><td>0.4</td><td>29.3</td><td>Symbols: MSI1, MEE70, ATMSI1 | Transducin/WD40 repeat-like superfamily protein | chr5:235</td><td>1 exon (s)</td><td>93681 - 94013</td><td>110 aa</td><td>chain -</td></tr>
<tr><td>26</td><td>FGENESH:26</td><td>AT5G41310.1</td><td>26.23</td><td>61</td><td>39</td><td>2</td><td>21</td><td>81</td><td>878</td><td>932</td><td>2.1</td><td>28.5</td><td>Symbols: | P-loop nucleoside triphosphate hydrolases superfamily protein with CH (Calponin Ho</td><td>2 exon (s)</td><td>94980 - 95755</td><td>164 aa</td><td>chain -</td></tr>
<tr><td>27</td><td>FGENESH:27</td><td>AT5G07720.1</td><td>78.71</td><td>465</td><td>90</td><td>5</td><td>1</td><td>464</td><td>1</td><td>457</td><td>0</td><td>766</td><td>Symbols: | Galactosyl transferase GMA12/MNN10 family protein | chr5:2455757-2457130 FORV</td><td>1 exon (s)</td><td>98093 - 99502</td><td>469 aa</td><td>chain +</td></tr>
<tr><td>28</td><td>FGENESH:28</td><td>AT5G61350.1</td><td>61.69</td><td>791</td><td>267</td><td>11</td><td>64</td><td>825</td><td>28</td><td>811</td><td>0</td><td>972</td><td>Symbols: | Protein kinase superfamily protein | chr5:24667973-24670501 FORWARD LENGTH=</td><td>3 exon (s)</td><td>101531 - 105626</td><td>1121 aa</td><td>chain -</td></tr>
<tr><td>29</td><td>FGENESH:29</td><td>AT4G29090.1</td><td>24.39</td><td>205</td><td>112</td><td>8</td><td>16</td><td>189</td><td>315</td><td>507</td><td>4e-05</td><td>44.7</td><td>Symbols: | Ribonuclease H-like superfamily protein | chr4:14333528-14335255 FORWARD LEN</td><td>4 exon (s)</td><td>107850 - 109317</td><td>286 aa</td><td>chain -</td></tr>
<tr><td>30</td><td>FGENESH:30</td><td>AT5G45140.1</td><td>40.91</td><td>22</td><td>13</td><td>0</td><td>19</td><td>40</td><td>395</td><td>416</td><td>2</td><td>25.8</td><td>Symbols: NRPC2 | nuclear RNA polymerase C2 | chr5:18247416-18257713 REVERSE LENGTH</td><td>2 exon (s)</td><td>110921 - 111682</td><td>71 aa</td><td>chain +</td></tr>
<tr><td>31</td><td>FGENESH:31</td><td>AT4G29035.1</td><td>28.26</td><td>138</td><td>84</td><td>3</td><td>5</td><td>142</td><td>11</td><td>133</td><td>4e-11</td><td>57.4</td><td>Symbols: | Plant self-incompatibility protein S1 family | chr4:14310315-14310770 FORWARD L</td><td>1 exon (s)</td><td>112025 - 112462</td><td>145 aa</td><td>chain -</td></tr>
<tr><td>32</td><td>FGENESH:32</td><td>AT3G26850.1</td><td>28.77</td><td>73</td><td>50</td><td>1</td><td>10</td><td>80</td><td>137</td><td>209</td><td>0.6</td><td>28.5</td><td>Symbols: | histone-lysine N-methyltransferases | chr3:9896541-9897415 REVERSE LENGTH=2</td><td>3 exon (s)</td><td>114471 - 116561</td><td>99 aa</td><td>chain +</td></tr>
<tr><td>33</td><td>FGENESH:33</td><td>AT4G29035.1</td><td>26.81</td><td>138</td><td>89</td><td>3</td><td>4</td><td>141</td><td>8</td><td>133</td><td>3e-11</td><td>58.2</td><td>Symbols: | Plant self-incompatibility protein S1 family | chr4:14310315-14310770 FORWARD L</td><td>1 exon (s)</td><td>117288 - 117758</td><td>156 aa</td><td>chain -</td></tr>
<tr><td>34</td><td>FGENESH:34</td><td>AT4G20520.1</td><td>40.35</td><td>57</td><td>33</td><td>1</td><td>213</td><td>269</td><td>17</td><td>72</td><td>5e-08</td><td>54.7</td><td>Symbols: | RNA binding;RNA-directed DNA polymerases | chr4:11045912-11047716 FORWAR</td><td>9 exon (s)</td><td>118645 - 122105</td><td>543 aa</td><td>chain -</td></tr>
<tr><td>35</td><td>FGENESH:35</td><td>AT4G23160.1</td><td>40.47</td><td>299</td><td>158</td><td>7</td><td>602</td><td>891</td><td>27</td><td>314</td><td>1e-59</td><td>223</td><td>Symbols: CRK8 | cysteine-rich RLK (RECEPTOR-like protein kinase) 8 | chr4:12129485-121340</td><td>7 exon (s)</td><td>122746 - 126900</td><td>1004 aa</td><td>chain -</td></tr>
<tr><td>36</td><td>FGENESH:36</td><td>AT3G42170.1</td><td>25.26</td><td>388</td><td>261</td><td>4</td><td>77</td><td>447</td><td>275</td><td>650</td><td>1e-32</td><td>134</td><td>Symbols: | BED zinc finger ;hAT family dimerisation domain | chr3:14321838-14323928 FORW</td><td>10 exon (s)</td><td>127118 - 131494</td><td>742 aa</td><td>chain -</td></tr>
<tr><td>37</td><td>FGENESH:37</td><td>AT3G18610.1</td><td>30.43</td><td>69</td><td>43</td><td>2</td><td>51</td><td>115</td><td>368</td><td>435</td><td>2.8</td><td>27.3</td><td>Symbols: PARLL1, ATNUC-L2, NUC-L2 | nucleolin like 2 | chr3:6404270-6407822 REVERSE L</td><td>3 exon (s)</td><td>132596 - 133601</td><td>122 aa</td><td>chain -</td></tr>
<tr><td>38</td><td>FGENESH:38</td><td>AT5G07910.1</td><td>73.08</td><td>156</td><td>42</td><td>0</td><td>1</td><td>156</td><td>70</td><td>225</td><td>5e-59</td><td>201</td><td>Symbols: | Leucine-rich repeat (LRR) family protein | chr5:2521937-2523769 REVERSE LENGT</td><td>10 exon (s)</td><td>134271 - 140232</td><td>640 aa</td><td>chain +</td></tr>
<tr><td>39</td><td>FGENESH:39</td><td>AT3G22550.1</td><td>33.93</td><td>56</td><td>35</td><td>2</td><td>38</td><td>92</td><td>45</td><td>99</td><td>3.5</td><td>28.1</td><td>Symbols: | Protein of unknown function (DUF581) | chr3:7991827-7992805 REVERSE LENGTH</td><td>4 exon (s)</td><td>140798 - 146288</td><td>214 aa</td><td>chain +</td></tr>
<tr><td>40</td><td>FGENESH:40</td><td></td><td></td><td></td><td></td><td></td><td></td><td></td><td></td><td></td><td></td><td></td><td></td><td>1 exon (s)</td><td>149038 - 149364</td><td>108 aa</td><td>chain +</td></tr>
<tr><td>41</td><td>FGENESH:41</td><td>AT1G48400.1</td><td>45.83</td><td>24</td><td>13</td><td>0</td><td>21</td><td>44</td><td>348</td><td>371</td><td>0.36</td><td>27.7</td><td>Symbols: | F-box/RNI-like/FBD-like domains-containing protein | chr1:17882136-17883855 REV</td><td>2 exon (s)</td><td>150859 - 151198</td><td>64 aa</td><td>chain +</td></tr>
<tr><td>42</td><td>FGENESH:42</td><td>AT3G52490.1</td><td>44.21</td><td>95</td><td>42</td><td>2</td><td>1</td><td>86</td><td>196</td><td>288</td><td>3e-15</td><td>70.9</td><td>Symbols: | Double Clp-N motif-containing P-loop nucleoside triphosphate hydrolases superfamily</td><td>2 exon (s)</td><td>151530 - 151824</td><td>90 aa</td><td>chain -</td></tr>
<tr><td>43</td><td>FGENESH:43</td><td>AT1G68890.1</td><td>58.33</td><td>24</td><td>8</td><td>1</td><td>63</td><td>86</td><td>390</td><td>411</td><td>3.4</td><td>26.2</td><td>Symbols: | magnesium ion binding;thiamin pyrophosphate binding;hydro-lyases;catalytics;2-succi</td><td>1 exon (s)</td><td>153166 - 153447</td><td>93 aa</td><td>chain +</td></tr>
<tr><td>44</td><td>FGENESH:44</td><td>AT2G45120.1</td><td>36.52</td><td>178</td><td>57</td><td>6</td><td>295</td><td>472</td><td>193</td><td>314</td><td>6e-23</td><td>99</td><td>Symbols: | C2H2-like zinc finger protein | chr2:18603680-18604624 FORWARD LENGTH=314</td><td>1 exon (s)</td><td>155774 - 157246</td><td>490 aa</td><td>chain +</td></tr>
<tr><td>45</td><td>FGENESH:45</td><td>AT3G23730.1</td><td>69.75</td><td>281</td><td>74</td><td>4</td><td>10</td><td>281</td><td>9</td><td>287</td><td>2e-145</td><td>414</td><td>Symbols: XTH16 | xyloglucan endotransglucosylase/hydrolase 16 | chr3:8550222-8551248 FORW</td><td>2 exon (s)</td><td>160175 - 161141</td><td>282 aa</td><td>chain -</td></tr>
<tr><td>46</td><td>FGENESH:46</td><td>AT1G48910.1</td><td>75.76</td><td>33</td><td>8</td><td>0</td><td>55</td><td>87</td><td>2</td><td>34</td><td>5e-11</td><td>59.3</td><td>Symbols: YUC10 | Flavin-containing monooxygenase family protein | chr1:18091681-18093774 F</td><td>3 exon (s)</td><td>162186 - 162907</td><td>127 aa</td><td>chain +</td></tr>
<tr><td>47</td><td>FGENESH:47</td><td>AT1G26570.1</td><td>76.47</td><td>85</td><td>20</td><td>0</td><td>123</td><td>207</td><td>397</td><td>481</td><td>6e-41</td><td>146</td><td>Symbols: UGD1, ATUGD1 | UDP-glucose dehydrogenase 1 | chr1:9182801-9184246 FORWARD</td><td>4 exon (s)</td><td>163412 - 165057</td><td>207 aa</td><td>chain +</td></tr>
<tr><td>48</td><td>FGENESH:48</td><td>AT4G25440.1</td><td>49.08</td><td>218</td><td>105</td><td>4</td><td>6</td><td>222</td><td>213</td><td>425</td><td>7e-61</td><td>199</td><td>Symbols: ZFWD1 | zinc finger WD40 repeat protein 1 | chr4:13007107-13009381 REVERSE LEN</td><td>2 exon (s)</td><td>166494 - 167325</td><td>231 aa</td><td>chain +</td></tr>
<tr><td>49</td><td>FGENESH:49</td><td>AT3G42170.1</td><td>36.36</td><td>88</td><td>56</td><td>0</td><td>97</td><td>184</td><td>588</td><td>675</td><td>1e-14</td><td>73.6</td><td>Symbols: | BED zinc finger ;hAT family dimerisation domain | chr3:14321838-14323928 FORW</td><td>4 exon (s)</td><td>170391 - 172322</td><td>280 aa</td><td>chain -</td></tr>
<tr><td>50</td><td>FGENESH:50</td><td>AT5G61340.1</td><td>40.64</td><td>342</td><td>172</td><td>9</td><td>1</td><td>342</td><td>1</td><td>311</td><td>9e-57</td><td>190</td><td>Symbols: | unknown protein; BEST Arabidopsis thaliana protein match is: unknown protein (TA</td><td>1 exon (s)</td><td>174800 - 175849</td><td>349 aa</td><td>chain +</td></tr>
<tr><td>51</td><td>FGENESH:51</td><td>AT1G18670.1</td><td>59.33</td><td>713</td><td>220</td><td>14</td><td>1</td><td>652</td><td>1</td><td>704</td><td>0</td><td>790</td><td>Symbols: IBS1 | Protein kinase superfamily protein | chr1:6427242-6430696 REVERSE LENGTH</td><td>13 exon (s)</td><td>176279 - 183786</td><td>1082 aa</td><td>chain -</td></tr>
<tr><td>52</td><td>FGENESH:52</td><td>AT1G77500.1</td><td>28.57</td><td>91</td><td>53</td><td>3</td><td>36</td><td>121</td><td>746</td><td>829</td><td>2.2</td><td>29.6</td><td>Symbols: | Protein of unknown function (DUF630 and DUF632) | chr1:29121753-29124937 FOR</td><td>2 exon (s)</td><td>185144 - 186053</td><td>296 aa</td><td>chain +</td></tr>
<tr><td>53</td><td>FGENESH:53</td><td>AT1G17980.1</td><td>55.87</td><td>775</td><td>228</td><td>13</td><td>20</td><td>757</td><td>5</td><td>702</td><td>0</td><td>794</td><td>Symbols: PAPS1 | poly(A) polymerase 1 | chr1:6187742-6191418 REVERSE LENGTH=713</td><td>13 exon (s)</td><td>188527 - 193396</td><td>781 aa</td><td>chain +</td></tr>
<tr><td>54</td><td>FGENESH:54</td><td>AT1G69360.1</td><td>32.42</td><td>293</td><td>173</td><td>9</td><td>643</td><td>914</td><td>597</td><td>885</td><td>8e-24</td><td>107</td><td>Symbols: | Plant protein of unknown function (DUF863) | chr1:26072520-26075420 REVERSE L</td><td>3 exon (s)</td><td>196637 - 199826</td><td>932 aa</td><td>chain +</td></tr>
<tr><td>55</td><td>FGENESH:55</td><td>AT2G37230.1</td><td>65.92</td><td>757</td><td>239</td><td>6</td><td>1</td><td>748</td><td>1</td><td>747</td><td>0</td><td>1042</td><td>Symbols: | Tetratricopeptide repeat (TPR)-like superfamily protein | chr2:15637177-15639450 R</td><td>1 exon (s)</td><td>202260 - 204563</td><td>767 aa</td><td>chain -</td></tr>
<tr><td>56</td><td>FGENESH:56</td><td>AT2G02955.1</td><td>38.87</td><td>674</td><td>348</td><td>10</td><td>14</td><td>634</td><td>3</td><td>665</td><td>1e-145</td><td>442</td><td>Symbols: MEE12 | maternal effect embryo arrest 12 | chr2:859592-861772 FORWARD LENGTH</td><td>4 exon (s)</td><td>204903 - 207506</td><td>645 aa</td><td>chain +</td></tr>
<tr><td>57</td><td>FGENESH:57</td><td>AT5G39380.1</td><td>41.18</td><td>153</td><td>72</td><td>5</td><td>820</td><td>971</td><td>372</td><td>507</td><td>4e-21</td><td>97.8</td><td>Symbols: | Plant calmodulin-binding protein-related | chr5:15759163-15760686 REVERSE LEN</td><td>1 exon (s)</td><td>208553 - 211495</td><td>980 aa</td><td>chain +</td></tr>
<tr><td>58</td><td>FGENESH:58</td><td>AT4G25440.1</td><td>40.04</td><td>452</td><td>230</td><td>10</td><td>7</td><td>455</td><td>12</td><td>425</td><td>2e-104</td><td>321</td><td>Symbols: ZFWD1 | zinc finger WD40 repeat protein 1 | chr4:13007107-13009381 REVERSE LEN</td><td>1 exon (s)</td><td>212337 - 213731</td><td>464 aa</td><td>chain +</td></tr>
<tr><td>59</td><td>FGENESH:59</td><td>AT5G04780.1</td><td>52.23</td><td>381</td><td>153</td><td>6</td><td>1</td><td>363</td><td>223</td><td>592</td><td>2e-126</td><td>381</td><td>Symbols: | Pentatricopeptide repeat (PPR) superfamily protein | chr5:1384540-1386447 FORWA</td><td>2 exon (s)</td><td>215158 - 216322</td><td>377 aa</td><td>chain +</td></tr>
<tr><td>60</td><td>FGENESH:60</td><td>AT2G47210.1</td><td>60.46</td><td>435</td><td>125</td><td>6</td><td>2</td><td>422</td><td>5</td><td>406</td><td>4e-177</td><td>515</td><td>Symbols: | myb-like transcription factor family protein | chr2:19379039-19382204 FORWARD L</td><td>19 exon (s)</td><td>216812 - 221806</td><td>662 aa</td><td>chain -</td></tr>
<tr><td>61</td><td>FGENESH:61</td><td>AT5G55820.1</td><td>34.38</td><td>64</td><td>39</td><td>1</td><td>2416</td><td>2479</td><td>1608</td><td>1668</td><td>0.51</td><td>35.8</td><td>Symbols: | CONTAINS InterPro DOMAIN/s: Inner centromere protein, ARK-binding region (Inte</td><td>11 exon (s)</td><td>225486 - 238481</td><td>3359 aa</td><td>chain -</td></tr>
<tr><td>62</td><td>FGENESH:62</td><td>AT5G51600.1</td><td>63.16</td><td>19</td><td>5</td><td>1</td><td>72</td><td>88</td><td>390</td><td>408</td><td>6.6</td><td>28.1</td><td>Symbols: PLE, ATMAP65-3, MAP65-3 | Microtubule associated protein (MAP65/ASE1) family</td><td>5 exon (s)</td><td>240659 - 245985</td><td>263 aa</td><td>chain -</td></tr>
<tr><td>63</td><td>FGENESH:63</td><td>AT5G07830.1</td><td>57.19</td><td>556</td><td>202</td><td>4</td><td>18</td><td>572</td><td>23</td><td>543</td><td>0</td><td>666</td><td>Symbols: AtGUS2, GUS2 | glucuronidase 2 | chr5:2504168-2506567 FORWARD LENGTH=543</td><td>9 exon (s)</td><td>246927 - 250067</td><td>572 aa</td><td>chain +</td></tr>
<tr><td>64</td><td>FGENESH:64</td><td>AT5G61220.1</td><td>58.14</td><td>86</td><td>36</td><td>0</td><td>7</td><td>92</td><td>2</td><td>87</td><td>3e-30</td><td>105</td><td>Symbols: | LYR family of Fe/S cluster biogenesis protein | chr5:24626057-24626320 REVERSE</td><td>1 exon (s)</td><td>250865 - 251152</td><td>95 aa</td><td>chain +</td></tr>
<tr><td>65</td><td>FGENESH:65</td><td>AT1G52360.2</td><td>32.56</td><td>43</td><td>29</td><td>0</td><td>26</td><td>68</td><td>248</td><td>290</td><td>0.12</td><td>30</td><td>Symbols: | Coatomer, beta' subunit | chr1:19499420-19505397 FORWARD LENGTH=970</td><td>1 exon (s)</td><td>252885 - 253157</td><td>90 aa</td><td>chain -</td></tr>
<tr><td>66</td><td>FGENESH:66</td><td>AT1G26600.1</td><td>29.06</td><td>117</td><td>65</td><td>5</td><td>15</td><td>119</td><td>10</td><td>120</td><td>6e-04</td><td>36.6</td><td>Symbols: CLE9 | CLAVATA3/ESR-RELATED 9 | chr1:9191684-9192046 FORWARD LENGTH</td><td>1 exon (s)</td><td>254042 - 254401</td><td>119 aa</td><td>chain -</td></tr>
<tr><td>67</td><td>FGENESH:67</td><td>AT1G48120.1</td><td>43.02</td><td>86</td><td>46</td><td>1</td><td>28</td><td>113</td><td>68</td><td>150</td><td>1e-09</td><td>60.1</td><td>Symbols: | hydrolases;protein serine/threonine phosphatases | chr1:17774238-17779624 REVERS</td><td>8 exon (s)</td><td>255841 - 258371</td><td>439 aa</td><td>chain +</td></tr>
<tr><td>68</td><td>FGENESH:68</td><td>AT1G49920.1</td><td>20.59</td><td>374</td><td>237</td><td>7</td><td>5</td><td>321</td><td>320</td><td>690</td><td>2e-07</td><td>52.8</td><td>Symbols: | MuDR family transposase | chr1:18481798-18484233 REVERSE LENGTH=785</td><td>5 exon (s)</td><td>258795 - 260230</td><td>348 aa</td><td>chain -</td></tr>
<tr><td>69</td><td>FGENESH:69</td><td>AT5G24300.1</td><td>30.23</td><td>43</td><td>24</td><td>2</td><td>67</td><td>106</td><td>201</td><td>240</td><td>7.4</td><td>26.2</td><td>Symbols: SSI1, SSI, ATSS1 | Glycogen/starch synthases, ADP-glucose type | chr5:8266934-82708</td><td>2 exon (s)</td><td>260422 - 260924</td><td>127 aa</td><td>chain +</td></tr>
<tr><td>70</td><td>FGENESH:70</td><td>AT4G25440.1</td><td>32.86</td><td>426</td><td>209</td><td>10</td><td>51</td><td>426</td><td>27</td><td>425</td><td>1e-65</td><td>219</td><td>Symbols: ZFWD1 | zinc finger WD40 repeat protein 1 | chr4:13007107-13009381 REVERSE LEN</td><td>4 exon (s)</td><td>261892 - 263718</td><td>435 aa</td><td>chain -</td></tr>
<tr><td>71</td><td>FGENESH:71</td><td>AT4G25440.1</td><td>47.58</td><td>330</td><td>169</td><td>3</td><td>155</td><td>483</td><td>98</td><td>424</td><td>2e-107</td><td>329</td><td>Symbols: ZFWD1 | zinc finger WD40 repeat protein 1 | chr4:13007107-13009381 REVERSE LEN</td><td>1 exon (s)</td><td>264473 - 265939</td><td>488 aa</td><td>chain -</td></tr>
<tr><td>72</td><td>FGENESH:72</td><td>AT1G43760.1</td><td>27.72</td><td>101</td><td>62</td><td>1</td><td>183</td><td>272</td><td>520</td><td>620</td><td>2e-06</td><td>49.3</td><td>Symbols: | DNAse I-like superfamily protein | chr1:16528880-16531065 REVERSE LENGTH=6</td><td>6 exon (s)</td><td>266797 - 268727</td><td>369 aa</td><td>chain +</td></tr>
<tr><td>73</td><td>FGENESH:73</td><td></td><td></td><td></td><td></td><td></td><td></td><td></td><td></td><td></td><td></td><td></td><td></td><td>2 exon (s)</td><td>269408 - 269710</td><td>63 aa</td><td>chain +</td></tr>
<tr><td>74</td><td>FGENESH:74</td><td>AT2G45240.1</td><td>80.05</td><td>396</td><td>75</td><td>1</td><td>4</td><td>399</td><td>7</td><td>398</td><td>0</td><td>696</td><td>Symbols: MAP1A | methionine aminopeptidase 1A | chr2:18656059-18658906 FORWARD LEN</td><td>16 exon (s)</td><td>270479 - 274734</td><td>399 aa</td><td>chain -</td></tr>
<tr><td>75</td><td>FGENESH:75</td><td>AT1G18700.4</td><td>46.8</td><td>656</td><td>260</td><td>14</td><td>116</td><td>719</td><td>97</td><td>715</td><td>0</td><td>544</td><td>Symbols: | DNAJ heat shock N-terminal domain-containing protein | chr1:6437591-6443046 FOR</td><td>19 exon (s)</td><td>275842 - 284593</td><td>719 aa</td><td>chain -</td></tr>
<tr><td>76</td><td>FGENESH:76</td><td>AT2G17920.1</td><td>21.82</td><td>165</td><td>127</td><td>1</td><td>5</td><td>169</td><td>37</td><td>199</td><td>2e-07</td><td>50.8</td><td>Symbols: | nucleic acid binding;zinc ion binding | chr2:7782808-7783731 FORWARD LENGTH=</td><td>2 exon (s)</td><td>288273 - 289274</td><td>266 aa</td><td>chain +</td></tr>
<tr><td>77</td><td>FGENESH:77</td><td>AT1G14740.1</td><td>50</td><td>26</td><td>13</td><td>0</td><td>27</td><td>52</td><td>80</td><td>105</td><td>0.81</td><td>26.9</td><td>Symbols: | Protein of unknown function (DUF1423) | chr1:5075450-5077732 REVERSE LENGT</td><td>2 exon (s)</td><td>289526 - 289789</td><td>69 aa</td><td>chain -</td></tr>
<tr><td>78</td><td>FGENESH:78</td><td>AT4G14350.1</td><td>35.71</td><td>42</td><td>26</td><td>1</td><td>32</td><td>73</td><td>266</td><td>306</td><td>4</td><td>26.2</td><td>Symbols: | AGC (cAMP-dependent, cGMP-dependent and protein kinase C) kinase family protei</td><td>2 exon (s)</td><td>290691 - 291553</td><td>104 aa</td><td>chain -</td></tr>
<tr><td>79</td><td>FGENESH:79</td><td>AT3G53640.1</td><td>41.67</td><td>36</td><td>19</td><td>1</td><td>33</td><td>68</td><td>409</td><td>442</td><td>0.93</td><td>28.1</td><td>Symbols: | Protein kinase superfamily protein | chr3:19887007-19888935 REVERSE LENGTH=</td><td>3 exon (s)</td><td>292903 - 293913</td><td>104 aa</td><td>chain +</td></tr>
<tr><td>80</td><td>FGENESH:80</td><td>AT3G45390.1</td><td>33.8</td><td>71</td><td>37</td><td>3</td><td>21</td><td>85</td><td>219</td><td>285</td><td>2.2</td><td>28.9</td><td>Symbols: | Concanavalin A-like lectin protein kinase family protein | chr3:16647921-16649974 R</td><td>7 exon (s)</td><td>296369 - 300354</td><td>204 aa</td><td>chain +</td></tr>
<tr><td>81</td><td>FGENESH:81</td><td>AT5G61430.1</td><td>55.62</td><td>356</td><td>108</td><td>12</td><td>7</td><td>336</td><td>5</td><td>336</td><td>3e-123</td><td>361</td><td>Symbols: ANAC100, ATNAC5, NAC100 | NAC domain containing protein 100 | chr5:24701328-</td><td>3 exon (s)</td><td>301799 - 302984</td><td>336 aa</td><td>chain -</td></tr>
<tr><td>82</td><td>FGENESH:82</td><td>AT3G23070.1</td><td>28.81</td><td>59</td><td>41</td><td>1</td><td>55</td><td>113</td><td>89</td><td>146</td><td>2.9</td><td>27.7</td><td>Symbols: ATCFM3A, CFM3A | CRM family member 3A | chr3:8203548-8207243 FORWARD L</td><td>4 exon (s)</td><td>305762 - 306511</td><td>139 aa</td><td>chain +</td></tr>
<tr><td>83</td><td>FGENESH:83</td><td>AT2G21800.2</td><td>26.67</td><td>75</td><td>48</td><td>1</td><td>35</td><td>102</td><td>372</td><td>446</td><td>0.67</td><td>29.3</td><td>Symbols: EME1A | essential meiotic endonuclease 1A | chr2:9298196-9301745 FORWARD LEN</td><td>3 exon (s)</td><td>307962 - 308473</td><td>135 aa</td><td>chain -</td></tr>
<tr><td>84</td><td>FGENESH:84</td><td>AT2G40850.1</td><td>46.67</td><td>30</td><td>16</td><td>0</td><td>113</td><td>142</td><td>118</td><td>147</td><td>2.2</td><td>28.9</td><td>Symbols: ATPI4K GAMMA 1, PI4K GAMMA 1 | phosphoinositide 4-kinase gamma 1 | chr2:170</td><td>3 exon (s)</td><td>308819 - 309688</td><td>213 aa</td><td>chain +</td></tr>
<tr><td>85</td><td>FGENESH:85</td><td>AT4G06534.1</td><td>29.63</td><td>81</td><td>55</td><td>2</td><td>23</td><td>101</td><td>195</td><td>275</td><td>2.2</td><td>27.3</td><td>Symbols: | unknown protein; FUNCTIONS IN: molecular_function unknown; INVOLVED IN: bi</td><td>2 exon (s)</td><td>313002 - 313552</td><td>109 aa</td><td>chain +</td></tr>
<tr><td>86</td><td>FGENESH:86</td><td>AT4G29090.1</td><td>37.21</td><td>129</td><td>80</td><td>1</td><td>102</td><td>229</td><td>38</td><td>166</td><td>4e-22</td><td>99.4</td><td>Symbols: | Ribonuclease H-like superfamily protein | chr4:14333528-14335255 FORWARD LEN</td><td>7 exon (s)</td><td>315058 - 320086</td><td>467 aa</td><td>chain -</td></tr>
<tr><td>87</td><td>FGENESH:87</td><td>AT4G10120.1</td><td>32.35</td><td>34</td><td>23</td><td>0</td><td>51</td><td>84</td><td>170</td><td>203</td><td>3.1</td><td>25.8</td><td>Symbols: ATSPS4F | Sucrose-phosphate synthase family protein | chr4:6315033-6319785 FORW</td><td>2 exon (s)</td><td>322266 - 322620</td><td>84 aa</td><td>chain +</td></tr>
<tr><td>88</td><td>FGENESH:88</td><td>AT1G21280.1</td><td>27.56</td><td>156</td><td>96</td><td>5</td><td>24</td><td>165</td><td>14</td><td>166</td><td>4e-12</td><td>62.8</td><td>Symbols: | CONTAINS InterPro DOMAIN/s: Retrotransposon gag protein (InterPro:IPR005162);</td><td>3 exon (s)</td><td>326943 - 328805</td><td>220 aa</td><td>chain +</td></tr>
<tr><td>89</td><td>FGENESH:89</td><td>AT3G25270.1</td><td>23.41</td><td>205</td><td>147</td><td>4</td><td>88</td><td>282</td><td>64</td><td>268</td><td>3e-11</td><td>63.2</td><td>Symbols: | Ribonuclease H-like superfamily protein | chr3:9203934-9204965 REVERSE LENGT</td><td>2 exon (s)</td><td>330708 - 331747</td><td>302 aa</td><td>chain -</td></tr>
<tr><td>90</td><td>FGENESH:90</td><td>AT1G43760.1</td><td>20.17</td><td>362</td><td>199</td><td>10</td><td>133</td><td>406</td><td>245</td><td>604</td><td>4e-09</td><td>59.3</td><td>Symbols: | DNAse I-like superfamily protein | chr1:16528880-16531065 REVERSE LENGTH=6</td><td>7 exon (s)</td><td>332121 - 335459</td><td>626 aa</td><td>chain -</td></tr>
<tr><td>91</td><td>FGENESH:91</td><td>AT1G18720.1</td><td>45.78</td><td>83</td><td>44</td><td>1</td><td>3</td><td>84</td><td>121</td><td>203</td><td>1e-17</td><td>74.7</td><td>Symbols: | Protein of unknown function (DUF962) | chr1:6458020-6459658 REVERSE LENGTH</td><td>3 exon (s)</td><td>337581 - 338019</td><td>88 aa</td><td>chain -</td></tr>
<tr><td>92</td><td>FGENESH:92</td><td>AT1G22670.1</td><td>39.47</td><td>38</td><td>23</td><td>0</td><td>48</td><td>85</td><td>126</td><td>163</td><td>0.49</td><td>28.9</td><td>Symbols: | Protease-associated (PA) RING/U-box zinc finger family protein | chr1:8021854-8023</td><td>2 exon (s)</td><td>338156 - 338737</td><td>109 aa</td><td>chain +</td></tr>
<tr><td>93</td><td>FGENESH:93</td><td>AT1G18720.1</td><td>59.5</td><td>200</td><td>78</td><td>1</td><td>1</td><td>197</td><td>1</td><td>200</td><td>5e-80</td><td>243</td><td>Symbols: | Protein of unknown function (DUF962) | chr1:6458020-6459658 REVERSE LENGTH</td><td>7 exon (s)</td><td>339311 - 341352</td><td>261 aa</td><td>chain -</td></tr>
<tr><td>94</td><td>FGENESH:94</td><td>AT3G47470.1</td><td>84.58</td><td>253</td><td>35</td><td>3</td><td>418</td><td>668</td><td>1</td><td>251</td><td>1e-149</td><td>437</td><td>Symbols: LHCA4, CAB4 | light-harvesting chlorophyll-protein complex I subunit A4 | chr3:17493</td><td>12 exon (s)</td><td>347052 - 350416</td><td>670 aa</td><td>chain +</td></tr>
<tr><td>95</td><td>FGENESH:95</td><td>AT1G74460.1</td><td>70.31</td><td>357</td><td>104</td><td>1</td><td>9</td><td>363</td><td>7</td><td>363</td><td>0</td><td>526</td><td>Symbols: | GDSL-like Lipase/Acylhydrolase superfamily protein | chr1:27988150-27989765 REV</td><td>4 exon (s)</td><td>351010 - 352550</td><td>373 aa</td><td>chain -</td></tr>
<tr><td>96</td><td>FGENESH:96</td><td>AT3G61990.1</td><td>44.44</td><td>36</td><td>17</td><td>2</td><td>6</td><td>41</td><td>26</td><td>58</td><td>2.2</td><td>28.9</td><td>Symbols: OMTF3 | S-adenosyl-L-methionine-dependent methyltransferases superfamily protein |</td><td>4 exon (s)</td><td>353199 - 354239</td><td>216 aa</td><td>chain -</td></tr>
<tr><td>97</td><td>FGENESH:97</td><td>AT1G74470.1</td><td>83.12</td><td>474</td><td>70</td><td>3</td><td>1</td><td>471</td><td>1</td><td>467</td><td>0</td><td>784</td><td>Symbols: | Pyridine nucleotide-disulphide oxidoreductase family protein | chr1:27991248-279928</td><td>2 exon (s)</td><td>355338 - 357349</td><td>471 aa</td><td>chain +</td></tr>
<tr><td>98</td><td>FGENESH:98</td><td>AT4G21270.1</td><td>50</td><td>26</td><td>12</td><td>1</td><td>54</td><td>79</td><td>9</td><td>33</td><td>0.73</td><td>29.3</td><td>Symbols: ATK1, KATA, KATAP | kinesin 1 | chr4:11329579-11333884 REVERSE LENGTH=79</td><td>2 exon (s)</td><td>360294 - 360976</td><td>128 aa</td><td>chain -</td></tr>
<tr><td>99</td><td>FGENESH:99</td><td>AT2G29510.1</td><td>36.49</td><td>74</td><td>32</td><td>3</td><td>86</td><td>159</td><td>338</td><td>396</td><td>0.93</td><td>30.8</td><td>Symbols: | Protein of unknown function (DUF3527) | chr2:12635231-12637838 FORWARD LEN</td><td>4 exon (s)</td><td>362129 - 363382</td><td>275 aa</td><td>chain +</td></tr>
<tr><td>100</td><td>FGENESH:100</td><td>ATMG01250.1</td><td>50</td><td>66</td><td>33</td><td>0</td><td>315</td><td>380</td><td>14</td><td>79</td><td>1e-12</td><td>67.4</td><td>Symbols: ORF102 | RNA-directed DNA polymerase (reverse transcriptase) | chrM:310514-31088</td><td>6 exon (s)</td><td>364547 - 368157</td><td>803 aa</td><td>chain -</td></tr>
<tr><td>101</td><td>FGENESH:101</td><td>AT1G16820.1</td><td>100</td><td>24</td><td>0</td><td>0</td><td>1</td><td>24</td><td>1</td><td>24</td><td>2e-09</td><td>49.7</td><td>Symbols: | vacuolar ATP synthase catalytic subunit-related / V-ATPase-related / vacuolar proton</td><td>2 exon (s)</td><td>371393 - 372444</td><td>67 aa</td><td>chain +</td></tr>
<tr><td>102</td><td>FGENESH:102</td><td>AT4G29090.1</td><td>34.72</td><td>72</td><td>38</td><td>3</td><td>292</td><td>361</td><td>7</td><td>71</td><td>0.15</td><td>34.3</td><td>Symbols: | Ribonuclease H-like superfamily protein | chr4:14333528-14335255 FORWARD LEN</td><td>6 exon (s)</td><td>372758 - 375428</td><td>452 aa</td><td>chain -</td></tr>
<tr><td>103</td><td>FGENESH:103</td><td>AT3G47520.1</td><td>83.65</td><td>312</td><td>51</td><td>0</td><td>84</td><td>395</td><td>80</td><td>391</td><td>0</td><td>542</td><td>Symbols: MDH | malate dehydrogenase | chr3:17513657-17514868 FORWARD LENGTH=403</td><td>1 exon (s)</td><td>376803 - 378020</td><td>405 aa</td><td>chain +</td></tr>
<tr><td>104</td><td>FGENESH:104</td><td>AT3G47530.1</td><td>54.05</td><td>309</td><td>96</td><td>6</td><td>118</td><td>385</td><td>288</td><td>591</td><td>8e-99</td><td>308</td><td>Symbols: | Pentatricopeptide repeat (PPR) superfamily protein | chr3:17517382-17519157 REVE</td><td>6 exon (s)</td><td>378338 - 380572</td><td>385 aa</td><td>chain -</td></tr>
<tr><td>105</td><td>FGENESH:105</td><td>AT5G62440.1</td><td>62.81</td><td>121</td><td>27</td><td>1</td><td>169</td><td>289</td><td>67</td><td>169</td><td>1e-50</td><td>169</td><td>Symbols: | Protein of unknown function (DUF3223) | chr5:25072620-25073917 REVERSE LEN</td><td>3 exon (s)</td><td>382146 - 383844</td><td>322 aa</td><td>chain -</td></tr>
<tr><td>106</td><td>FGENESH:106</td><td>AT3G47550.3</td><td>59.39</td><td>293</td><td>108</td><td>7</td><td>109</td><td>395</td><td>1</td><td>288</td><td>2e-93</td><td>285</td><td>Symbols: | RING/FYVE/PHD zinc finger superfamily protein | chr3:17523841-17525278 FORW</td><td>10 exon (s)</td><td>387318 - 391464</td><td>401 aa</td><td>chain +</td></tr>
<tr><td>107</td><td>FGENESH:107</td><td>AT2G23100.1</td><td>43.59</td><td>39</td><td>17</td><td>2</td><td>18</td><td>56</td><td>453</td><td>486</td><td>8.2</td><td>26.6</td><td>Symbols: | Cysteine/Histidine-rich C1 domain family protein | chr2:9837564-9839699 REVERSE</td><td>1 exon (s)</td><td>393404 - 393892</td><td>162 aa</td><td>chain -</td></tr>
<tr><td>108</td><td>FGENESH:108</td><td>AT2G30490.1</td><td>86.05</td><td>430</td><td>56</td><td>1</td><td>1</td><td>426</td><td>73</td><td>502</td><td>0</td><td>775</td><td>Symbols: ATC4H, C4H, CYP73A5, REF3 | cinnamate-4-hydroxylase | chr2:12993861-12995683</td><td>4 exon (s)</td><td>395055 - 398669</td><td>489 aa</td><td>chain -</td></tr>
<tr><td>109</td><td>FGENESH:109</td><td>AT3G27640.1</td><td>41.38</td><td>29</td><td>17</td><td>0</td><td>2</td><td>30</td><td>507</td><td>535</td><td>0.65</td><td>26.9</td><td>Symbols: | Transducin/WD40 repeat-like superfamily protein | chr3:10232307-10235467 FORW</td><td>2 exon (s)</td><td>400665 - 400880</td><td>65 aa</td><td>chain +</td></tr>
<tr><td>110</td><td>FGENESH:110</td><td>AT1G49870.1</td><td>33.33</td><td>54</td><td>27</td><td>1</td><td>53</td><td>97</td><td>232</td><td>285</td><td>0.46</td><td>29.3</td><td>Symbols: | unknown protein; LOCATED IN: chloroplast; EXPRESSED IN: 10 plant structures; E</td><td>2 exon (s)</td><td>401156 - 401581</td><td>108 aa</td><td>chain -</td></tr>
<tr><td>111</td><td>FGENESH:111</td><td>AT5G09910.1</td><td>31.03</td><td>58</td><td>33</td><td>1</td><td>11</td><td>68</td><td>169</td><td>219</td><td>1.5</td><td>26.9</td><td>Symbols: | Ras-related small GTP-binding family protein | chr5:3093272-3094932 FORWARD L</td><td>2 exon (s)</td><td>402477 - 403142</td><td>87 aa</td><td>chain +</td></tr>
<tr><td>112</td><td>FGENESH:112</td><td>AT3G24255.1</td><td>26.48</td><td>321</td><td>157</td><td>8</td><td>7</td><td>249</td><td>9</td><td>328</td><td>3e-22</td><td>97.4</td><td>Symbols: | RNA-directed DNA polymerase (reverse transcriptase)-related family protein | chr3:87</td><td>3 exon (s)</td><td>404945 - 405987</td><td>302 aa</td><td>chain -</td></tr>
<tr><td>113</td><td>FGENESH:113</td><td>AT1G02960.2</td><td>33.33</td><td>39</td><td>24</td><td>1</td><td>4</td><td>40</td><td>335</td><td>373</td><td>1.9</td><td>25.8</td><td>Symbols: | unknown protein; BEST Arabidopsis thaliana protein match is: unknown protein (TA</td><td>2 exon (s)</td><td>407359 - 407618</td><td>63 aa</td><td>chain +</td></tr>
<tr><td>114</td><td>FGENESH:114</td><td>AT5G15280.1</td><td>30.43</td><td>69</td><td>42</td><td>2</td><td>64</td><td>126</td><td>49</td><td>117</td><td>0.44</td><td>32.3</td><td>Symbols: | Pentatricopeptide repeat (PPR) superfamily protein | chr5:4962293-4965976 FORWA</td><td>3 exon (s)</td><td>414457 - 415977</td><td>347 aa</td><td>chain +</td></tr>
<tr><td>115</td><td>FGENESH:115</td><td>AT5G58300.1</td><td>68.53</td><td>626</td><td>188</td><td>4</td><td>8</td><td>632</td><td>27</td><td>644</td><td>0</td><td>856</td><td>Symbols: | Leucine-rich repeat protein kinase family protein | chr5:23572821-23574871 FORWA</td><td>9 exon (s)</td><td>416446 - 422288</td><td>1258 aa</td><td>chain -</td></tr>
</table>

116	FGENESH:116	AT3G31430.1	25.53	94	69	1	72	165	214	306	1e-04	43.1	Symbols: | unknown protein; FUNCTIONS IN: molecular_function unknown; INVOLVED IN: bi	6 exon (s)	423248 - 426351	310 aa	chain -
117	FGENESH:117	AT1G73390.1	27.63	76	53	1	56	129	341	416	3e-04	41.2	Symbols: | Endosomal targeting BRO1-like domain-containing protein | chr1:27591079-2759414	4 exon (s)	427508 - 429029	226 aa	chain +
118	FGENESH:118	AT3G10860.1	68.75	32	10	0	4	35	6	37	7e-12	55.8	Symbols: | Cytochrome b-c1 complex, subunit 8 protein | chr3:3399815-3400514 FORWARD LE	2 exon (s)	431499 - 431809	60 aa	chain -
119	FGENESH:119	AT3G55010.1	34.88	43	27	1	47	89	242	283	4.2	25.8	Symbols: ATPURM, PUR5 | phosphoribosylformylglycinamidine cyclo-ligase, chloroplast / phosp	2 exon (s)	432604 - 433000	90 aa	chain +
120	FGENESH:120	AT5G10860.1	80.58	206	37	2	1	205	1	204	3e-114	332	Symbols: | Cystathionine beta-synthase (CBS) family protein | chr5:3429173-3430142 REVERSE	6 exon (s)	434049 - 436228	310 aa	chain +
121	FGENESH:121	AT5G40090.1	38.46	39	19	2	65	103	246	279	1.8	27.3	Symbols: | Disease resistance protein (TIR-NBS class) | chr5:16042115-16043494 REVERSE LE	1 exon (s)	437638 - 437958	106 aa	chain -
122	FGENESH:122	AT2G38610.1	62.55	267	80	7	74	327	27	286	5e-103	310	Symbols: | RNA-binding KH domain-containing protein | chr2:16147552-16149638 REVERSE L	8 exon (s)	438590 - 441069	388 aa	chain -
123	FGENESH:123	AT2G24880.1	26.32	38	28	0	231	268	48	85	2.5	28.1	Symbols: | Plant self-incompatibility protein S1 family | chr2:10590554-10590862 REVERSE LE	4 exon (s)	442171 - 443856	336 aa	chain -
124	FGENESH:124	AT1G01930.1	42.31	26	15	0	27	52	318	343	1.2	27.3	Symbols: | zinc finger protein-related | chr1:320041-322809 REVERSE LENGTH=580	1 exon (s)	444202 - 444477	91 aa	chain -
125	FGENESH:125	AT3G17740.1	38.89	36	21	1	2	36	953	988	0.082	30.4	Symbols: | unknown protein; FUNCTIONS IN: molecular_function unknown; INVOLVED IN: bi	2 exon (s)	446649 - 446944	82 aa	chain +
126	FGENESH:126	AT3G29785.1	29.35	92	61	2	3	93	8	96	3e-07	44.7	Symbols: | unknown protein; Has 90 Blast hits to 90 proteins in 7 species: Archae - 0; Bacteria - (	1 exon (s)	448030 - 448338	102 aa	chain +
127	FGENESH:127	AT2G33580.1	43.01	93	49	2	66	158	333	421	1e-15	75.1	Symbols: | Protein kinase superfamily protein | chr2:14219848-14221842 REVERSE LENGTH=6	2 exon (s)	448897 - 449514	179 aa	chain +
128	FGENESH:128	AT3G25890.1	35.94	345	146	14	16	337	40	332	3e-43	154	Symbols: | Integrase-type DNA-binding superfamily protein | chr3:9476052-9477050 FORWARD	1 exon (s)	451686 - 452699	337 aa	chain +
129	FGENESH:129	AT1G73660.1	24.26	136	83	6	27	162	899	1014	0.13	32.3	Symbols: | protein tyrosine kinase family protein | chr1:27692247-27696718 REVERSE LENGTH	2 exon (s)	453816 - 454562	183 aa	chain +
130	FGENESH:130	AT1G68560.1	39.63	217	103	5	1	190	307	522	2e-36	136	Symbols: ATXYL1, XYL1, TRG1 | alpha-xylosidase 1 | chr1:25734435-25737897 REVERSE LE	1 exon (s)	454749 - 455321	190 aa	chain -
131	FGENESH:131	AT3G45940.1	63.95	172	59	2	77	248	119	287	1e-68	228	Symbols: | Glycosyl hydrolases family 31 protein | chr3:16886226-16889171 REVERSE LENGT	2 exon (s)	456271 - 457199	248 aa	chain -
132	FGENESH:132	AT1G68560.1	53.57	112	33	3	26	130	71	170	3e-28	110	Symbols: ATXYL1, XYL1, TRG1 | alpha-xylosidase 1 | chr1:25734435-25737897 REVERSE LE	2 exon (s)	457628 - 458654	131 aa	chain +
133	FGENESH:133	AT1G68560.1	61.14	229	84	2	1	229	332	555	2e-96	304	Symbols: ATXYL1, XYL1, TRG1 | alpha-xylosidase 1 | chr1:25734435-25737897 REVERSE LE	1 exon (s)	459076 - 459765	229 aa	chain +
134	FGENESH:134	AT3G12820.1	50	36	12	1	54	83	73	108	0.058	32.3	Symbols: AtMYB10, MYB10 | myb domain protein 10 | chr3:4074328-4075614 REVERSE LEN(	3 exon (s)	460082 - 460835	135 aa	chain +
135	FGENESH:135	AT5G54440.1	68.23	1108	339	8	226	1327	159	1259	0	1571	Symbols: CLUB, AtTRS130 | CLUB | chr5:22100056-22107695 FORWARD LENGTH=1259	28 exon (s)	461471 - 473411	1569 aa	chain +
136	FGENESH:136	AT1G14990.2	70.27	111	33	0	1	111	1	111	3e-57	177	Symbols: | unknown protein; FUNCTIONS IN: molecular_function unknown; INVOLVED IN: bi	4 exon (s)	473592 - 474489	147 aa	chain -
137	FGENESH:137	AT5G62270.2	63.42	380	111	4	83	436	7	384	4e-144	421	Symbols: | FUNCTIONS IN: molecular_function unknown; INVOLVED IN: biological_process u	5 exon (s)	474858 - 477784	437 aa	chain +
138	FGENESH:138	AT2G26000.2	66.74	451	130	5	72	511	3	444	0	632	Symbols: BRIZ2 | zinc finger (C3HC4-type RING finger) family protein | chr2:11082403-110851	13 exon (s)	478624 - 483110	541 aa	chain -
139	FGENESH:139	AT3G21750.1	34.38	32	21	0	67	98	140	171	1	27.7	Symbols: UGT71B1 | UDP-glucosyl transferase 71B1 | chr3:7664565-7665986 FORWARD LEN(	1 exon (s)	483541 - 483837	98 aa	chain +
140	FGENESH:140	AT4G23160.1	38.39	534	313	7	668	1191	74	601	1e-101	355	Symbols: CRK8 | cysteine-rich RLK (RECEPTOR-like protein kinase) 8 | chr4:12129485-121340	5 exon (s)	484531 - 490774	1529 aa	chain -
141	FGENESH:141	AT4G39670.1	52.94	102	34	2	84	185	25	112	4e-25	102	Symbols: | Glycolipid transfer protein (GLTP) family protein | chr4:18410172-18410861 FORWA	7 exon (s)	492216 - 496769	328 aa	chain -
142	FGENESH:142	AT1G68585.1	29.71	138	35	2	47	184	27	102	3e-11	58.5	Symbols: | unknown protein; Has 23 Blast hits to 23 proteins in 6 species: Archae - 0; Bacteria - (	4 exon (s)	499437 - 501078	206 aa	chain +
143	FGENESH:143	AT2G26800.2	41.38	58	29	2	50	102	92	149	0.002	37	Symbols: | Aldolase superfamily protein | chr2:11429192-11432290 REVERSE LENGTH=468	3 exon (s)	501666 - 502397	138 aa	chain -
144	FGENESH:144	AT4G23160.1	36.74	362	142	6	1	275	193	554	8e-62	214	Symbols: CRK8 | cysteine-rich RLK (RECEPTOR-like protein kinase) 8 | chr4:12129485-121340	3 exon (s)	502843 - 504019	312 aa	chain -
145	FGENESH:145	AT1G21280.1	36.21	58	35	1	52	107	19	76	0.024	35.4	Symbols: | CONTAINS InterPro DOMAIN/s: Retrotransposon gag protein (InterPro:IPR005162);	3 exon (s)	505387 - 506477	299 aa	chain -
146	FGENESH:146	AT4G29090.1	36.51	126	77	1	195	317	20	145	4e-15	78.6	Symbols: | Ribonuclease H-like superfamily protein | chr4:14333528-14335255 FORWARD LEN	8 exon (s)	507074 - 511955	618 aa	chain +
147	FGENESH:147	AT3G25855.1	52.53	99	42	2	15	111	6	101	1e-29	107	Symbols: | Copper transport protein family | chr3:9459608-9460267 REVERSE LENGTH=112	4 exon (s)	514230 - 516069	163 aa	chain +
148	FGENESH:148	AT3G24255.1	40	45	27	0	69	113	9	53	5e-04	40.4	Symbols: | RNA-directed DNA polymerase (reverse transcriptase)-related family protein | chr3:87	2 exon (s)	518610 - 519393	218 aa	chain -
149	FGENESH:149	AT1G79190.1	32.31	65	43	1	77	141	885	948	0.67	29.6	Symbols: | ARM repeat superfamily protein | chr1:29788639-29794116 FORWARD LENGTH=1	1 exon (s)	519832 - 520287	151 aa	chain -
150	FGENESH:150	AT3G27110.1	42.5	40	17	1	10	43	139	178	0.5	29.6	Symbols: | Peptidase family M48 family protein | chr3:9998006-9999892 FORWARD LENGTH=	1 exon (s)	520818 - 521201	127 aa	chain +
151	FGENESH:151	AT1G12700.1	50	30	13	1	37	64	296	325	0.15	29.6	Symbols: | ATP binding;nucleic acid binding;helicases | chr1:4323722-4326227 REVERSE LEN(	4 exon (s)	521986 - 523051	86 aa	chain -
152	FGENESH:152	AT2G19240.1	39.39	33	20	0	22	54	730	762	4.6	25.8	Symbols: | Ypt/Rab-GAP domain of gyp1p superfamily protein | chr2:8349048-8352562 REVERS	2 exon (s)	524565 - 525430	99 aa	chain -
153	FGENESH:153	AT1G33750.1	24.67	150	75	6	22	161	77	198	0.1	32.7	Symbols: | Terpenoid cyclases/Protein prenyltransferases superfamily protein | chr1:12233769-12	1 exon (s)	526553 - 527074	173 aa	chain +
154	FGENESH:154	AT3G25840.1	52.78	809	302	16	206	990	175	927	0	710	Symbols: | Protein kinase superfamily protein | chr3:9452993-9457446 REVERSE LENGTH=93	14 exon (s)	529185 - 536504	1087 aa	chain +
155	FGENESH:155	AT1G13330.1	68.95	219	68	0	107	325	6	224	8e-111	325	Symbols: AHP2 | Arabidopsis Hop2 homolog | chr1:4568008-4569410 FORWARD LENGTH=22(	8 exon (s)	537922 - 540946	327 aa	chain -
156	FGENESH:156	AT2G32000.1	63.63	888	216	7	46	850	2	865	0	1099	Symbols: | DNA topoisomerase, type IA, core | chr2:13615999-13621563 REVERSE LENGTH=8	16 exon (s)	541793 - 548048	850 aa	chain +
157	FGENESH:157	AT1G68590.1	59.35	155	61	1	18	172	13	165	1e-56	178	Symbols: | Ribosomal protein PSRP-3/Ycf65 | chr1:25757593-25758169 REVERSE LENGTH=1	2 exon (s)	548804 - 549392	172 aa	chain -
158	FGENESH:158	AT1G25480.1	60.22	455	158	7	13	462	3	439	3e-180	524	Symbols: | Aluminium activated malate transporter family protein | chr1:8948467-8950876 FORV	6 exon (s)	550199 - 553809	564 aa	chain +
159	FGENESH:159	AT5G44450.1	25.2	127	91	3	11	134	41	166	0.42	32	Symbols: | methyltransferases | chr5:17914360-17916017 REVERSE LENGTH=334	4 exon (s)	555386 - 558790	351 aa	chain -
160	FGENESH:160	AT4G20520.1	41.07	56	32	1	278	333	28	82	1e-06	50.8	Symbols: | RNA binding;RNA-directed DNA polymerases | chr4:11045912-11047716 FORWARI	11 exon (s)	559946 - 564249	619 aa	chain -
161	FGENESH:161	AT3G25800.1	91.26	595	43	1	18	612	1	586	0	1119	Symbols: PDF1, PR 65, PP2AA2 | protein phosphatase 2A subunit A2 | chr3:9422822-9425783 R	12 exon (s)	564992 - 569056	612 aa	chain +
162	FGENESH:162	AT1G61640.1	24.62	65	43	1	5	63	227	291	0.55	28.1	Symbols: | Protein kinase superfamily protein | chr1:22746629-22749053 REVERSE LENGTH=6	2 exon (s)	570352 - 571997	82 aa	chain +
163	FGENESH:163	AT1G14870.1	52.78	144	65	1	56	196	9	152	9e-56	176	Symbols: PCR2 | PLANT CADMIUM RESISTANCE 2 | chr1:5128591-5129458 REVERSE LEN(	4 exon (s)	572704 - 573952	198 aa	chain -
164	FGENESH:164													2 exon (s)	574709 - 576575	99 aa	chain -
165	FGENESH:165	AT1G25520.1	70.67	75	22	0	156	230	109	183	4e-30	115	Symbols: | Uncharacterized protein family (UPF0016) | chr1:8962324-8964173 FORWARD LEN	8 exon (s)	576871 - 580075	309 aa	chain +
166	FGENESH:166	AT1G68660.1	43.08	130	34	3	16	145	38	127	2e-21	89.4	Symbols: | Ribosomal protein L12/ ATP-dependent Clp protease adaptor protein ClpS family prot	5 exon (s)	580284 - 581820	288 aa	chain -
167	FGENESH:167	AT1G29700.1	65.09	338	105	3	1	336	13	339	6e-150	431	Symbols: | Metallo-hydrolase/oxidoreductase superfamily protein | chr1:10385196-10386906 RE	8 exon (s)	582794 - 585002	359 aa	chain +
168	FGENESH:168	AT1G25550.1	48.56	348	103	11	1	334	9	294	3e-86	268	Symbols: | myb-like transcription factor family protein | chr1:8976644-8977942 FORWARD LEN	4 exon (s)	585384 - 586937	391 aa	chain -
169	FGENESH:169	AT3G25270.1	29.63	54	38	0	231	284	18	71	0.004	38.1	Symbols: | Ribonuclease H-like superfamily protein | chr3:9203934-9204965 REVERSE LENGT	3 exon (s)	588247 - 589954	285 aa	chain -
170	FGENESH:170	AT2G36835.1	32.43	37	24	1	1	36	1	37	1.1	25.4	Symbols: | unknown protein; FUNCTIONS IN: molecular_function unknown; INVOLVED IN: bi	2 exon (s)	591207 - 591880	41 aa	chain +
171	FGENESH:171	AT3G42170.1	29.61	206	142	3	84	288	168	371	5e-24	105	Symbols: | BED zinc finger ;hAT family dimerisation domain | chr3:14321838-14323928 FORW	8 exon (s)	594076 - 598069	479 aa	chain -
172	FGENESH:172	AT1G48490.1	37.78	45	23	2	57	101	544	583	0.49	30	Symbols: | Protein kinase superfamily protein | chr1:17922345-17928597 REVERSE LENGTH=1	2 exon (s)	598286 - 598785	141 aa	chain -
173	FGENESH:173	AT1G11300.1	34.69	49	30	1	240	288	1570	1616	1.6	30.4	Symbols: | protein serine/threonine kinases;protein kinases;ATP binding;sugar binding;kinases;ca	3 exon (s)	599910 - 603041	303 aa	chain +
174	FGENESH:174	AT1G13290.1	69.11	246	60	6	35	278	12	243	9e-113	333	Symbols: DOT5, WIP6 | C2H2-like zinc finger protein | chr1:4550366-4551527 REVERSE LENG	2 exon (s)	603739 - 605054	330 aa	chain +
175	FGENESH:175	AT1G13270.1	73.45	339	88	2	33	370	30	367	0	518	Symbols: MAP1C, MAP1B | methionine aminopeptidase 1B | chr1:4544999-4547155 FORWARD	10 exon (s)	612751 - 615709	372 aa	chain -
176	FGENESH:176	AT4G17410.3	27.81	392	206	10	4	348	1	362	3e-36	140	Symbols: | DWNN domain, a CCHC-type zinc finger | chr4:9716360-9721642 FORWARD LEN(	1 exon (s)	616286 - 617377	363 aa	chain +
177	FGENESH:177	AT5G07740.1	35.29	51	32	1	18	67	622	672	0.069	30.8	Symbols: | actin binding | chr5:2459076-2466580 REVERSE LENGTH=1649	2 exon (s)	619195 - 619557	89 aa	chain +
178	FGENESH:178	AT5G47090.1	60.91	197	53	4	1	196	137	310	1e-58	189	Symbols: | unknown protein; CONTAINS InterPro DOMAIN/s: Protein of unknown function DUI	2 exon (s)	619765 - 620450	197 aa	chain -
179	FGENESH:179	AT5G23880.1	73.54	650	157	3	1	649	75	710	0	1000	Symbols: EMB1265, CPSF100, ESP5, ATCPSF100 | cleavage and polyadenylation specificity fac	17 exon (s)	624058 - 629302	669 aa	chain -
180	FGENESH:180	AT2G16360.1	87.1	62	8	0	40	101	42	103	3e-32	113	Symbols: | Ribosomal protein S25 family protein | chr2:7076713-7077366 REVERSE LENGTH=	4 exon (s)	629917 - 632739	144 aa	chain +
181	FGENESH:181	AT1G70590.1	31.15	61	38	1	85	145	266	322	1.7	28.5	Symbols: | F-box family protein | chr1:26618403-26620159 FORWARD LENGTH=351	3 exon (s)	634321 - 636676	160 aa	chain -
182	FGENESH:182	AT1G13260.1	59.04	354	116	10	7	346	3	341	4e-126	369	Symbols: RAV1, EDF4 | related to ABI3/VP1 1 | chr1:4542386-4543420 FORWARD LENGTH=	1 exon (s)	638635 - 639687	350 aa	chain -
183	FGENESH:183	AT5G26860.1	30.11	93	53	3	53	139	210	296	0.18	32.3	Symbols: LON_ARA_ARA, LON1 | lon protease 1 | chr5:9451183-9456631 FORWARD LENGT	4 exon (s)	641693 - 643538	219 aa	chain -
184	FGENESH:184	AT5G23770.2	26.21	103	61	3	28	121	404	500	0.5	30	Symbols: DUF8 | DOMAIN OF UNKNOWN FUNCTION 724 8 | chr5:8014513-8016755 REVEF	3 exon (s)	644994 - 646622	156 aa	chain -
185	FGENESH:185	AT3G25270.1	55.56	27	12	0	4	30	17	43	4e-04	40	Symbols: | Ribonuclease H-like superfamily protein | chr3:9203934-9204965 REVERSE LENGT	2 exon (s)	647204 - 647850	195 aa	chain +
186	FGENESH:186	ATCG00770.1	36.84	38	24	0	1	38	38	75	1.4	25.8	Symbols: RPS8 | ribosomal protein S8 | chrC:80068-80472 REVERSE LENGTH=134	2 exon (s)	649641 - 649979	67 aa	chain -
187	FGENESH:187	AT1G43760.1	31.11	90	62	0	6	95	532	621	2e-09	59.7	Symbols: | DNAse I-like superfamily protein | chr1:16528880-16531065 REVERSE LENGTH=6	6 exon (s)	658066 - 661152	452 aa	chain +
188	FGENESH:188	AT1G43760.1	41.79	67	39	0	73	139	532	598	2e-09	55.5	Symbols: | DNAse I-like superfamily protein | chr1:16528880-16531065 REVERSE LENGTH=6	3 exon (s)	661558 - 662396	144 aa	chain +
189	FGENESH:189	ATMG01250.1	53.45	58	27	0	93	150	23	80	8e-12	63.5	Symbols: ORF102 | RNA-directed DNA polymerase (reverse transcriptase) | chrM:310514-31088	7 exon (s)	663960 - 667408	564 aa	chain +
190	FGENESH:190	AT1G25580.1	55.65	460	177	12	39	486	5	449	3e-158	462	Symbols: SOG1, ANAC008 | NAC (No Apical Meristem) domain transcriptional regulator superf	7 exon (s)	668498 - 672022	525 aa	chain -
191	FGENESH:191	AT2G33620.1	34.04	47	25	1	13	59	218	258	0.13	30.4	Symbols: | AT hook motif DNA-binding family protein | chr2:14234749-14236563 FORWARD L	1 exon (s)	672848 - 673162	104 aa	chain +
192	FGENESH:192	AT3G09650.1	50	18	9	0	24	41	252	269	0.23	28.5	Symbols: HCF152, CRM3 | Tetratricopeptide repeat (TPR)-like superfamily protein | chr3:29587(	2 exon (s)	674426 - 674977	68 aa	chain +
193	FGENESH:193	AT1G55915.1	32.39	71	43	3	35	100	52	122	0.85	29.6	Symbols: | zinc ion binding | chr1:20907663-20909429 FORWARD LENGTH=404	3 exon (s)	675673 - 676827	178 aa	chain -
194	FGENESH:194	AT1G68820.1	55.06	425	125	7	110	470	1	423	2e-142	432	Symbols: | Transmembrane Fragile-X-F-associated protein | chr1:25865852-25868800 FORWAR	16 exon (s)	678221 - 687606	812 aa	chain -
195	FGENESH:195	AT2G32930.2	35.71	42	21	1	2	37	15	56	2.1	25.4	Symbols: ZFN2 | zinc finger nuclease 2 | chr2:13965814-13968419 REVERSE LENGTH=467	2 exon (s)	688960 - 689344	63 aa	chain +
196	FGENESH:196	AT5G36870.1	39.13	23	14	0	20	42	407	429	7.7	25.4	Symbols: | CONTAINS InterPro DOMAIN/s: WLM (InterPro:IPR013536), PUB domain (InterPro	3 exon (s)	694381 - 696826	99 aa	chain +
197	FGENESH:197	AT1G27540.1	38.46	39	24	0	25	63	277	315	0.14	30	Symbols: | Protein of unknown function (DUF295) | chr1:9566608-9567849 REVERSE LENGTH	1 exon (s)	698524 - 698826	100 aa	chain +
198	FGENESH:198	AT1G73610.1	25.58	86	51	2	43	128	7	79	3.4	28.9	Symbols: | GDSL-like Lipase/Acylhydrolase superfamily protein | chr1:27678377-27679807 FOR	4 exon (s)	699843 - 701898	269 aa	chain -
199	FGENESH:199	AT2G20930.1	26.23	61	38	2	23	82	41	95	1.2	26.9	Symbols: | SNARE-like superfamily protein | chr2:9000790-9001656 REVERSE LENGTH=140	3 exon (s)	702657 - 703641	84 aa	chain +
200	FGENESH:200	AT3G51480.1	29.63	54	37	1	55	107	281	334	0.57	28.9	Symbols: ATGLR3.6, GLR3.6 | glutamate receptor 3.6 | chr3:19101256-19104681 FORWARD LI	2 exon (s)	706358 - 706749	110 aa	chain +
201	FGENESH:201	ATCG00160.1	26.92	156	92	4	95	247	62	198	0.49	32	Symbols: RPS2 | ribosomal protein S2 | chrC:15013-15723 REVERSE LENGTH=236	4 exon (s)	709352 - 712335	417 aa	chain +
202	FGENESH:202	AT1G09240.1	27.27	77	52	1	52	124	184	260	3.1	28.1	Symbols: NAS3, ATNAS3 | nicotianamine synthase 3 | chr1:2984950-2985912 FORWARD LEN(	3 exon (s)	713907 - 715041	181 aa	chain +
203	FGENESH:203	AT2G36240.1	41.03	39	22	1	29	67	18	55	0.18	29.3	Symbols: | pentatricopeptide (PPR) repeat-containing protein | chr2:15195663-15197156 FORW	2 exon (s)	717271 - 718480	79 aa	chain -
204	FGENESH:204	AT1G01370.1	28.09	89	44	2	10	90	45	121	0.47	30.4	Symbols: HTR12, CENH3 | Histone superfamily protein | chr1:143773-145400 FORWARD LEN(	6 exon (s)	718803 - 721461	193 aa	chain +
205	FGENESH:205	AT1G72150.1	28.07	57	31	1	132	178	315	371	0.3	31.6	Symbols: PATL1 | PATELLIN 1 | chr1:27148558-27150652 FORWARD LENGTH=573	4 exon (s)	723971 - 727119	191 aa	chain +
206	FGENESH:206	AT4G10590.2	27.14	70	43	2	32	93	269	338	3.4	28.9	Symbols: UBP10 | ubiquitin-specific protease 10 | chr4:6538919-6543272 REVERSE LENGTH=9	2 exon (s)	727507 - 728634	262 aa	chain -
207	FGENESH:207	AT1G68810.1	62.41	290	73	7	105	379	100	368	4e-102	310	Symbols: | basic helix-loop-helix (bHLH) DNA-binding superfamily protein | chr1:25861289-258	2 exon (s)	731243 - 733997	384 aa	chain +
208	FGENESH:208	AT3G25120.1	36.36	44	23	1	18	61	139	177	0.18	28.5	Symbols: | Mitochondrial import inner membrane translocase subunit Tim17/Tim22/Tim23 famil	2 exon (s)	735271 - 735799	65 aa	chain +
209	FGENESH:209	AT3G24255.1	45.83	48	24	1	46	93	90	135	5e-06	43.9	Symbols: | RNA-directed DNA polymerase (reverse transcriptase)-related family protein | chr3:87	2 exon (s)	737803 - 738601	115 aa	chain +
210	FGENESH:210	AT3G09300.1	45	40	21	1	62	100	43	82	0.55	29.3	Symbols: ORP3B | OSBP(oxysterol binding protein)-related protein 3B | chr3:2858068-2860462 F	2 exon (s)	740722 - 741111	119 aa	chain +
211	FGENESH:211	AT1G04300.3	68.42	19	2	1	59	73	669	687	0.36	30	Symbols: | TRAF-like superfamily protein | chr1:1148818-1153895 REVERSE LENGTH=1082	3 exon (s)	741204 - 742035	132 aa	chain -
212	FGENESH:212	AT4G15545.1	22.33	103	63	2	139	230	69	165	5.6	27.7	Symbols: | unknown protein; BEST Arabidopsis thaliana protein match is: unknown protein (TAI	4 exon (s)	744179 - 746772	247 aa	chain +
213	FGENESH:213	AT2G02870.1	40	40	23	1	46	84	105	144	0.55	29.3	Symbols: | Galactose oxidase/kelch repeat superfamily protein | chr2:838378-839781 FORWARD	2 exon (s)	748118 - 748764	119 aa	chain -
214	FGENESH:214	AT3G18550.1	33.46	260	121	7	24	235	46	301	5e-26	107	Symbols: BRC1, TCP18, ATTCP18 | TCP family transcription factor | chr3:6383769-6385604 FC	2 exon (s)	749466 - 750932	335 aa	chain +
215	FGENESH:215	AT1G49000.1	66.67	30	10	0	139	168	104	133	3e-08	50.1	Symbols: | unknown protein; FUNCTIONS IN: molecular_function unknown; INVOLVED IN: bi	1 exon (s)	752967 - 753494	175 aa	chain +
216	FGENESH:216	AT3G25680.1	42.46	617	279	13	58	664	1	551	9e-139	422	Symbols: | FUNCTIONS IN: molecular_function unknown; INVOLVED IN: biological_process u	11 exon (s)	753764 - 757267	691 aa	chain -
217	FGENESH:217	AT1G56590.1	38.71	31	18	1	69	98	344	374	4	26.2	Symbols: ZIP4 | Clathrin adaptor complexes medium subunit family protein | chr1:21202250-212(	1 exon (s)	758769 - 759095	108 aa	chain +
218	FGENESH:218	AT1G26100.1	56.03	116	43	3	4	114	124	236	3e-28	104	Symbols: | Cytochrome b561/ferric reductase transmembrane protein family | chr1:9022716-9024	1 exon (s)	759867 - 760211	114 aa	chain -
219	FGENESH:219	AT1G26100.1	47.62	42	16	1	1	36	1	42	0.095	32	Symbols: | Cytochrome b561/ferric reductase transmembrane protein family | chr1:9022716-9024	4 exon (s)	760421 - 761731	155 aa	chain -
220	FGENESH:220	AT1G52990.1	28.71	101	71	1	4	104	66	165	0.003	35.4	Symbols: | thioredoxin family protein | chr1:19740503-19743449 REVERSE LENGTH=313	1 exon (s)	761984 - 762325	113 aa	chain -
221	FGENESH:221	AT4G05320.2	99.78	457	1	0	24	480	1	457	0	903	Symbols: UBQ10 | polyubiquitin 10 | chr4:2718559-2719932 FORWARD LENGTH=457	1 exon (s)	763940 - 765382	480 aa	chain -
222	FGENESH:222	AT4G00210.1	37.5	40	22	2	73	110	23	61	1.9	27.3	Symbols: LBD31 | LOB domain-containing protein 31 | chr4:85693-86888 REVERSE LENGTH=	3 exon (s)	766847 - 768926	118 aa	chain +
223	FGENESH:223	AT1G63240.1	47.06	17	9	0	34	50	12	28	3.5	25	Symbols: | unknown protein; Has 460 Blast hits to 413 proteins in 106 species: Archae - 5; Bacte	2 exon (s)	769071 - 770484	70 aa	chain -
224	FGENESH:224	AT5G15740.1	74	477	119	1	34	505	23	499	0	754	Symbols: | O-fucosyltransferase family protein | chr5:5134788-5136956 REVERSE LENGTH=50	9 exon (s)	771004 - 773817	532 aa	chain +
225	FGENESH:225	AT3G24255.1	28.85	156	108	2	882	1036	8	161	5e-14	76.3	Symbols: | RNA-directed DNA polymerase (reverse transcriptase)-related family protein | chr3:87	12 exon (s)	776761 - 784995	1039 aa	chain -
226	FGENESH:226	AT1G73880.1	51.38	327	143	6	9	330	11	326	1e-104	321	Symbols: UGT89B1 | UDP-glucosyl transferase 89B1 | chr1:27785143-27786564 FORWARD LE	2 exon (s)	789249 - 790492	408 aa	chain +
227	FGENESH:227													3 exon (s)	790976 - 792802	282 aa	chain +
228	FGENESH:228	AT3G42660.1	58.35	910	327	12	1	882	1	886	0	1024	Symbols: | transducin family protein / WD-40 repeat family protein | chr3:14751280-14755701 F	12 exon (s)	793253 - 798035	967 aa	chain -
229	FGENESH:229	ATMG01250.1	61.82	55	21	0	596	650	26	80	1e-14	72.4	Symbols: ORF102 | RNA-directed DNA polymerase (reverse transcriptase) | chrM:310514-31088	11 exon (s)	798911 - 802137	703 aa	chain +
230	FGENESH:230	AT5G65005.1	28.32	113	75	2	127	233	47	159	3e-06	47	Symbols: | Polynucleotidyl transferase, ribonuclease H-like superfamily protein | chr5:25967824-	5 exon (s)	802348 - 803663	248 aa	chain +
231	FGENESH:231	AT1G26310.1	26.67	105	68	4	22	119	29	131	1.2	28.1	Symbols: CAL, CAL1, AGL10 | K-box region and MADS-box transcription factor family protein	1 exon (s)	807126 - 807491	121 aa	chain +
232	FGENESH:232	AT1G73990.1	68.6	242	74	1	1	242	437	676	8e-110	334	Symbols: SPPA, SPPA1 | signal peptide peptidase | chr1:27824465-27828807 FORWARD LENG	5 exon (s)	808467 - 809802	242 aa	chain -
233	FGENESH:233	AT4G23160.1	42.33	489	275	2	750	1232	43	530	1e-126	425	Symbols: CRK8 | cysteine-rich RLK (RECEPTOR-like protein kinase) 8 | chr4:12129485-121340	4 exon (s)	810975 - 815994	1292 aa	chain +

<table>
<tr><td>234</td><td>FGENESH:234</td><td>AT1G73990.1</td><td>62.61</td><td>329</td><td>117</td><td>4</td><td>277</td><td>601</td><td>31</td><td>357</td><td>1e-137</td><td>420</td><td>Symbols: SPPA, SPPA1 | signal peptide peptidase | chr1:27824465-27828807 FORWARD LENG</td><td>9 exon (s)</td><td>816343</td><td>- 820151</td><td>610 aa</td><td>chain -</td></tr>
<tr><td>235</td><td>FGENESH:235</td><td>AT1G17970.1</td><td>34.76</td><td>328</td><td>173</td><td>11</td><td>1</td><td>303</td><td>56</td><td>367</td><td>2e-44</td><td>157</td><td>Symbols: | RING/U-box superfamily protein | chr1:6185032-6187202 FORWARD LENGTH=36</td><td>4 exon (s)</td><td>826622</td><td>- 828795</td><td>304 aa</td><td>chain +</td></tr>
<tr><td>236</td><td>FGENESH:236</td><td>AT2G01190.1</td><td>36.36</td><td>55</td><td>22</td><td>3</td><td>22</td><td>76</td><td>639</td><td>680</td><td>0.073</td><td>32</td><td>Symbols: | Octicosapeptide/Phox/Bem1p family protein | chr2:115023-117296 FORWARD LEN</td><td>1 exon (s)</td><td>830183</td><td>- 830563</td><td>126 aa</td><td>chain -</td></tr>
</table>

Supplementary Table S3. Genes identified by FGENESH prediction of the 53.5-kb region of Scaffold 208 in the vicinity of the mapped *D* locus and by BLAST analysis of the TAIR database. The gene of interest is highlighted in yellow.

	TAIR ID	%ID	Length	e-value	bitscore	Description	Region	aa	strand
1	AT1G18650.1	68.89	90	4e-38	138	Encodes a member of the X8-GPI family of proteins. It localizes to the plasmodesmata and is predicted to bind callose. protein_coding PLASMODESMATA CALLOSE-BINDING PROTEIN 3 (PDCB3) PLASMODESMATA CALLOSE-BINDING PROTEIN 3 (PDCB3)	654 - 4897	436 aa	chain -
2	AT4G19710.2	50	30	0.59	29.3	Encodes a bifunctional aspartate kinase/homoserine dehydrogenase. These two activities catalyze the first and the third steps toward the synthesis of the essential amino acids threonine, isoleucine and methionine. protein_coding ASPARTATE KINASE-HOMOSERINE DEHYDROGENASE II (AK-HSDH II) ASPARTATE KINASE-HOMOSERINE DEHYDROGENASE (AK-HSDH);ASPARTATE KINASE-HOMOSERINE DEHYDROGENASE II (AK-HSDH II)	5224 - 6099	124 aa	chain +
3	AT5G07990.1	61.69	509	0	656	Required for flavonoid 3' hydroxylase activity. protein_coding TRANSPARENT TESTA 7 (TT7) TRANSPARENT TESTA 7 (TT7); (D501);CYTOCHROME P450 75B1 (CYP75B1)	7127 - 9095	521 aa	chain -
4	AT5G11040.1	26.47	68	1.3	29.3	Encodes a tethering factor required for cell plate biogenesis. protein_coding (TRS120) (TRS120); (AtTRS120)	10278 - 13980	178 aa	chain +
5	AT1G11100.2	26.95	141	3.1	27.3	SNF2 domain-containing protein / helicase domain-containing protein / zinc finger protein-related; FUNCTIONS IN: helicase activity, DNA binding, zinc ion binding, nucleic acid binding, ATP binding; helicase domain-containing protein / zinc finger protein-related (TAIR:AT1G61140.1). protein_coding	15246 - 15611	121 aa	chain -
6	AT5G03040.1	30.77	39	0.76	29.3	IQ-domain 2 (iqd2); FUNCTIONS IN: calmodulin binding; INVOLVED IN: biological_process unknown; LOCATED IN: plasma membrane	17050 - 17603	138 aa	chain -
7	AT2G21590.1	29.33	75	0.89	26.9	Encodes the large subunit of ADP-glucose pyrophosphorylase, the enzyme which catalyzes the first and limiting step in starch biosynthesis. The large subunit plays a regulatory role whereas the small subunit (ApS) is the catalytic isoform. Four isoforms of the large subunit (ApL1-4) have been described. protein_coding (APL4) (APL4)	20388 - 20612	74 aa	chain -
8	AT5G07980.1	37.78	540	7e-72	265	dentin sialophosphoprotein-related; BEST Arabidopsis thaliana protein match is: unknown protein (TAIR:AT5G07940.2); Has 1336 Blast hits to 783 proteins in 174 species: Archae - 4; Bacteria - 291; Metazoa - 248; Fungi - 128; Plants - 141; Viruses - 0; Other Eukaryotes - 524 (source: NCBI BLink). protein_coding	22686 - 27964	1366 aa	chain +
9	AT5G07950.1	41	239	1e-47	164	unknown protein; FUNCTIONS IN: molecular_function unknown; INVOLVED IN: biological_process unknown; LOCATED IN: chloroplast; EXPRESSED IN: 20 plant structures; EXPRESSED DURING: 9 growth stages; Has 30201 Blast hits to 17322 proteins in 780 species: Archae - 12; Bacteria - 1396; Metazoa - 17338; Fungi - 3422; Plants - 5037; Viruses - 0; Other Eukaryotes - 2996 (source: NCBI BLink). protein_coding	28131 - 30004	314 aa	chain -
10	AT5G07960.1	55.71	140	6e-47	150	unknown protein; CONTAINS InterPro DOMAIN/s: Uncharacterised protein family UPF0139 (InterPro:IPR005351); Has 193 Blast hits to 193 proteins in 75 species: Archae - 0; Bacteria - 0; Metazoa - 130; Fungi - 0; Plants - 52; Viruses - 0; Other Eukaryotes - 11 (source: NCBI BLink). protein_coding	30347 - 32172	145 aa	chain +
11	AT5G39510.1	74.87	199	1e-104	319	Encodes a member of SNARE gene family. Homologous with yeast VTI1 and is involved in vesicle transport. Mutant alleles such as sgr4/zig are defective in the shoots response to gravity resulting in a zigzag growth pattern of the stem. Involved in protein trafficking to lytic vacuoles. Can conditionally substitute VTI12 in protein storage vacuole trafficking when plants are devoid of VTI12. protein_coding SHOOT GRAVITROPSIM 4 (SGR4) (ZIG1); (VTI1A);SHOOT GRAVITROPSIM 4 (SGR4); (ZIG);VESICLE TRANSPORT V-SNARE 11 (ATVTI11); (ATVTI1A);VESICLE TRANSPORT V-SNARE 11 (VTI11)	33577 - 37672	637 aa	chain -
12	ATMG00810.1	43.14	51	5e-10	55.8	hypothetical protein protein_coding (ORF240B) (ORF240B)	43051 - 44019	161 aa	chain -
13	AT1G77470.1	29.17	72	0.24	29.6	Encodes a protein with high homology to the Replication Factor C, Subunit 3 (RFC3) of yeast and other eukaryotes. rfc3 mutants are hypersensitive to salicylic acid and exhibit enhanced induction of PR genes and resistance against virulent oomycete Hyaloperonospora arabidopsidis Noco2. The enhanced pathogen resistance in the mutant is NPR1-independent.	46323 - 47364	108 aa	chain +
14	AT5G07920.1	71.64	744	0	1096	diacylglycerol kinase protein_coding DIACYLGLYCEROL KINASE1 (DGK1) DIACYLGLYCEROL KINASE 1 (ATDGK1);DIACYLGLYCEROL KINASE1 (DGK1)	48975 - 52821	726 aa	chain +
